# Robust Lanthanoid Picolinate-Based Coordination Polymers for Luminescence and Sensing Applications

**DOI:** 10.1021/acs.inorgchem.1c01229

**Published:** 2021-07-07

**Authors:** Verónica Jornet-Mollá, Chris Dreessen, Francisco M. Romero

**Affiliations:** Instituto de Ciencia Molecular, Universitat de València, P.O. Box 22085, 46071 València, Spain

## Abstract

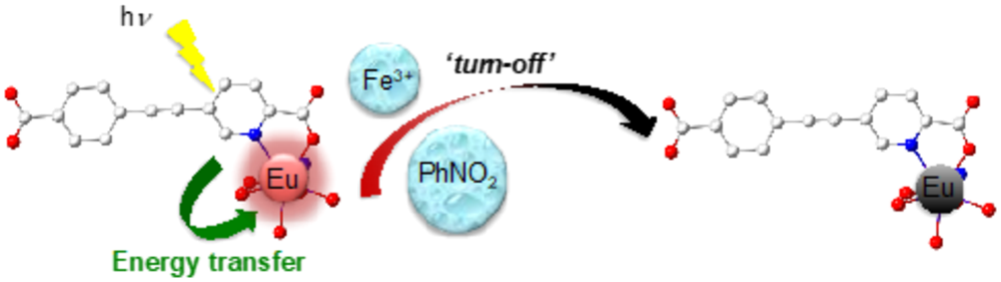

Picolinate-based segmented dianionic ligands **L**_**1**_^**2–**^ (5-((4-carboxyphenyl)ethynyl)picolinate) and **L**_**2**_^**2–**^ (5,5′-(ethyne-1,2-diyl)dipicolinate) have been used in the synthesis of the highly robust and luminescent europium(III) coordination polymers [(CH_3_)_2_NH_2_][Eu(H_2_O)_2_(**L**_**1**_)_2_] (**1**) and [(CH_3_)_2_NH_2_][Eu(**L**_**2**_)_2_]·H_2_O·CH_3_COOH (**2**). Both **1** and **2** exhibit high selectivity for detection of nitroaromatic compounds since they act as quenchers of the Eu^3+^ emission. Stern–Volmer plots, using nitrobenzene as a quencher, yielded values of *K*_SV_ = 150 M^–1^ and 160 M^–1^ for **1** and **2**, respectively. Luminescence studies in the presence of different metal ions indicate a high selectivity for Fe^3+^ detection, with *K*_SV_ values of 471 M^–1^ and 706 M^–1^ for **1** and **2**, respectively. Both **1** and **2** possess extremely robust extended structures, leading to emissive properties that are stable in a wide pH range.

## Abstract 2

Luminescent europium(III) coordination polymers exhibiting a remarkable robustness have been prepared from ethynyl-bridged picolinate ligands. These compounds can selectively detect nitrobenzene and Fe^3+^ ions in aqueous medium through luminescence quenching. Furthermore, they present a high stability over a wide pH range that allows their practical use in harsh conditions.

## Introduction

Coordination polymers (CPs) are crystalline materials formed by the self-assembly of metallic centers and multicoordinating organic molecules as bridging ligands through metal–ligand bonds.^1−5^ The common interest in these compounds has been driven by their promising applications in fields such as ion exchange,^6^ gas adsorption and separation processes,^7−10^ drug delivery,^11^ luminescence and sensing,^12−14^ and catalysis.^15−17^

The design of luminescent sensors remains a hot topic due to its high demand in a wide variety of fields, such as analytical chemistry, clinical biochemistry, and environmental science. Supramolecular interactions of luminescent CPs with the analyte may increase or decrease the luminescence response, resulting in a turn-on or turn-off display, respectively.^18−22^ The use of coordination polymers as fluorescent probes relies mostly on fluorescence quenching.^23,24^ Different types of analytes can be detected by this method, and there are already many fluorescent molecular sensors commercially available; however, their selectivity, limit of detection, and toxicity should be improved.

Early detection of metal ions such as Pb^2+^, Cd^2+^, Hg^2+^, Al^3+^, and Fe^3+^ is highly recommended, due to their accumulation as pollutants in water. Fe(III) is one of the most common and relevant components in the Earth’s crust and is also present in biological systems, playing an important role in metabolic processes. Its presence in an appropriate amount is essential for regular growth; nevertheless, an excess of iron(III) can lead to health problems. Recently, Ln-CPs have been found to be promising candidates in this regard, their main drawback being their poor stability in aqueous medium. The first example of a europium(III) fluorescent sensor for Fe^3+^ detection was developed by Dang et al. in 2012. Nonetheless, the selective quenching mechanism consisted of cation exchange between the original metal ion and Fe^3+^ cations rather than in a specific interaction.^25^ Later, more Eu-CPs have been applied for the sensing of Fe^3+^ ions, and Table S1 contains some examples with corresponding *K*_SV_ (Stern–Volmer constant) and limit of detection (LOD) values. The quenching mechanisms are based on cation exchange between Fe^3+^ and Eu^3+^, competitive absorption of excitation energy, and the interaction between Fe^3+^ and the organic ligand.^26−33^

Nitroaromatic compounds (NACs) represent also serious sources of pollution of soils and groundwater, mainly because of their high toxicity. Such materials are also frequently used as explosive materials in terrorism. Highly sensitive and efficient materials able to detect trace amounts of NACs in water are thus in strong demand. It was not until 2015 that Ln-CPs were applied as fluorescent sensors for NACs detection, exhibiting quenching in the presence of nitro explosives with estimated *K*_SV_ values among the highest known for CPs.^34^Table S2 contains a list of selected metallic complexes used for detection of nitrobenzene (chosen as a representative nitroaromatic compound), together with the *K*_SV_ and LOD achieved. Zn(II)-CPs are those which present the best parameters (higher *K*_SV_ and lower LOD) in this context.

Most metal–organic frameworks are however easily hydrolyzed. In order to construct stable complexes, strong coordination bonds are needed, and several strategies are currently being developed for this purpose.^35^ One is the combination of carboxylate-based ligands and high-valent metal ions. Alternatively, soft azolate ligands in conjunction with soft divalent metal ions have also been used.

Another approach is focused on chelating anionic ligands, which is an almost unexplored alternative to that based on high-valent complexes. There are only a few examples related to tetraanionic bisbidentate dioxidobenzenedicarboxylate ligands^36−38^ and catecholates.^39^ In this context, the picolinate (pic) ligand is a promising chelating anionic fragment for the design of robust CPs with high stability. On the other hand, ethynylene bridges have been incorporated in the design of some organic linkers because they provide rigidity, linear connectivity, and electron delocalization.^40,41^ Despite the fact that the picolinate ligand is a universal chelating anionic entity, only a few segmented polytopic picolinate ligands have been reported^42−45^ and their use in metal–organic frameworks is unexplored. Further, picolinate ligands are known to act as photosensitizers, transferring the absorbed energy in the UV region to lanthanoid cations, resulting in an enhanced lanthanoid luminescence (antenna effect).^46^ For example, a 10^4^-fold enhancement of Tb(III) luminescence by using dipicolinate ligands as photosensitizers has been reported.^47^

**Chart 1:**
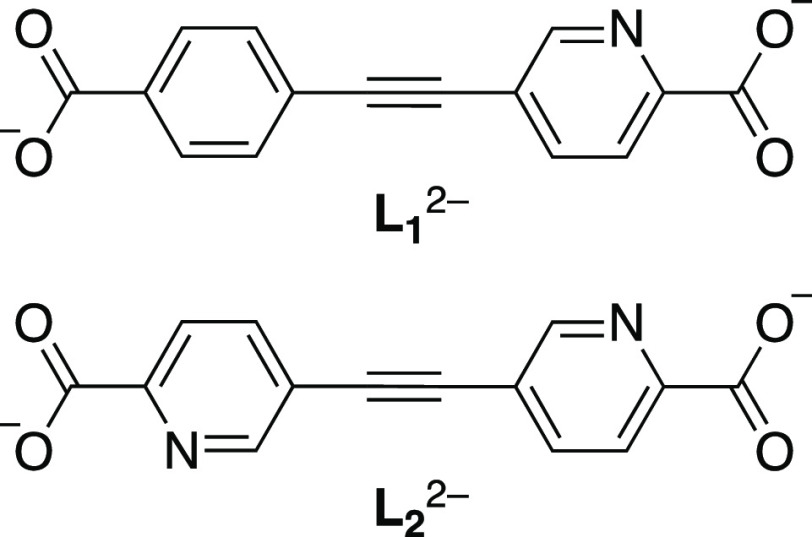
Segmented Picolinate-Based Ligands Used in This Work

We report herein on the synthesis, structural characterization, and optical properties of highly robust europium(III) coordination polymers based on the segmented ligands (Chart 1) 5-((4-carboxyphenyl)ethynyl)picolinate (**L**_**1**_^2–^) and 5,5′-(ethyne-1,2-diyl)dipicolinate (**L**_**2**_^2–^). **L**_**1**_^2–^ is a heteroditopic ligand containing a picolinate chelating unit and a benzoate anion connected by an ethynylene linker. **L**_**2**_^2–^ is homoditopic, the ethynylene rigid spacer connecting two picolinate chelating units. Comparison is given on the emission properties of these Eu^3+^ compounds and their use in sensing of metal ions and nitroaromatic pollutants under extreme pH conditions.

## Experimental Section

All chemicals and solvents were used as received. The synthesis of ligands **L**_**1**_^2–^ and **L**_**2**_^2–^ was previously described.^48^

### Synthesis of [(CH_3_)_2_NH_2_][Eu(H_2_O)_2_(**L**_**1**_)_2_] (**1**)

A solution of Eu(NO_3_)_3_·6H_2_O (11.15 mg, 0.025 mmol) and 5-((4- carboxyphenyl)ethynyl)picolinic acid (13.35 mg, 0.05 mmol) in 2.5 mL of DMF/H_2_O (3:2 ratio) was placed in a glass vial and stirred for 30 min. Next, two drops of nitric acid (65%, aq.) were added, and the resulting suspension was placed in an oven. The mixture was heated at 130 °C for 3 days and then cooled to room temperature at a cooling rate of 0.2 K·min^–1^. Colorless spearhead-shaped crystals were obtained, filtered, and air-dried to give compound **1**. Yield: 15.1 mg (79%). Elem anal. calcd for C_32_H_26_EuN_3_O_10_: C, 50.27; H, 3.43; N, 5.50. Found: C, 49.86; H, 3.38; N, 5.36. IR (cm^–1^): 3097, 3075, 3019, 2963, 2801, 2221, 1631, 1587, 1373, 1356, 1244, 782, 724, 693, 662, 627, 560, 400.

### Synthesis of [(CH_3_)NH_2_][Eu(**L**_**2**_)_2_]·H_2_O·CH_3_COOH (**2**)

A mixture of Eu(NO_3_)_3_·6H_2_O (24.9 mg, 0.056 mmol) and 5,5′-(ethyne-1,2-diyl)dipicolinic acid (15 mg, 0.056 mmol) in H_2_O (0.5 mL) and DMF (4 mL) was stirred in a glass vial for 30 min until a solution was formed. Then, acetic acid (1100 μL) was added, and the mixture was placed in an oven at 130 °C for 3 days. A cooling rate of 0.2 K·min^–1^ was applied to cool down the sample to room temperature. After the liquid was decanted, colorless aggregated crystals of **2** were obtained. The crystals were washed with water and dried in the air. Yield: 14.3 mg (60%). Elem. anal. calcd for C_32_H_26_EuN_5_O_11_·2H_2_O: C, 45.51; H, 3.58; N, 8.29. Found: C, 45.22; H, 3.11; N, 8.29. IR (cm^–1^): 3383, 3033, 2761, 2461, 1606, 1556, 1361, 1244, 1033, 806, 700, 661, 628, 389.

### Single-Crystal X-ray Diffraction

Suitable crystals of **1** and **2** were coated with paratone N oil, fixed on a small fiber loop, and mounted on an Oxford Diffraction Supernova diffractometer equipped with a graphite-monochromated Enhance Mo X-ray source (λ = 0.71073 Å) at 120 K. The data collection routines, unit cell refinements, and data processing were carried out using the CrysAlis software package.^49^ The structures were solved using SHELXT 2018/2 via the WinGX graphical interface^50^ and refined using SHELXL-2018/3.^51^ All non-hydrogen atoms were refined anisotropically (DELU and SIMU restraints were applied to C and O atoms of the acetic acid molecule present in **2** to allow their anisotropic refinement). H atoms on carbon atoms were included at calculated positions and refined with a riding model with relative isotropic displacement parameters. Instead, H atoms on solvent molecules and amine H atoms on dimethylammonium cations were found in Fourier difference maps, except for H atoms of O1W in compound **2**. CCDC 2068840 and CCDC 2068875 contain the supplementary crystallographic data for **1** and **2**, respectively. These data are provided free of charge by The Cambridge Crystallographic Data Centre.

### Powder X-ray Diffraction (PXRD)

PXRD measurements of compounds **1** and **2** were collected using Cu Kα radiation (λ = 1.54056 Å) at room temperature and in a 2θ range from 2 to 40°. Polycrystalline samples were lightly ground in an agate mortar and filled into a 0.5 mm borosilicate capillary prior to being mounted and aligned on an Empyrean PANalytical powder diffractometer. For pH-dependent measurements, the samples were soaked in aqueous solutions with pH values ranging from 1 to 14 for 2 h. Then, the samples were filtered and air-dried prior to their analysis. Simulated diffractograms were obtained from single-crystal X-ray data using the CrystalDiffract software.

### Absorption Spectroscopy and Photoluminescent Properties

UV–visible absorption spectra were recorded at room temperature for complexes **1** and **2** and for the ligands in their acid forms on a JASCO V-670 absorption spectrometer. Also, the absorption spectra of solvent samples and 0.01 M aqueous solutions of different metal ions were registered at room temperature. Measurements were performed in the 240–1000 nm range. Photoluminescent data of both complexes in aqueous suspensions were obtained with a Varian Cary Eclipse spectrometer, in quartz cuvettes (1 cm path length). For pH-sensing measurements, the solid materials (1 mg) were vigorously dispersed in aqueous solutions (1 mL) at different pH values. Emission spectra were registered after 2 h of sample preparation. The range of study was 540–720 nm with excitation wavelengths of 344 and 340 nm for compounds **1** and **2**, respectively. For titration experiments, 25 mM or 50 mM solution of Fe(NO_3_)_3_ in water (for compounds **1** and **2**, respectively) or 0.1 M nitrobenzene in EtOH were incrementally added to a cuvette containing 1 mg of the sensor in 1 mL of Milli-Q water, and emission spectra were recorded at each point.

The photoluminescence characteristics were studied in the solid state using a Xe lamp coupled to a monochromator as the excitation source and an integrated sphere coupled to a spectrometer (Hamamatsu C9920-02 with a Hamamatsu PMA-11 optical detector) in order to quantitatively determine the quantum yield for complexes **1** and **2**. Luminescence decay measurements were measured using an Edinburgh FLS 1000 spectrometer setup and Fluoracle software. The samples were excited by a 375 nm laser (CNI MLL-III-375-100 mW) at a frequency of 20 Hz. The luminescence was collected via two monochromators at 614 nm with a bandwidth of 1 nm in the multi-channel-scaling mode.

### Other Characterization Techniques

IR transmission measurements of both complexes were performed directly from the powdered samples at room temperature in a FT-IR spectrometer (Bruker, alpha II) equipped with an attenuated total reflection (ATR) accessory in the range 400–4000 cm^–1^. C, H, and N elemental analyses were performed using a CE INSTRUMENTS 1110 EA elemental analyzer (SCSIE, Universitat de València). Thermogravimetric analyses of salts **1** and **2** were performed on a Mettler-Toledo TGA/SDTA/851e apparatus under N_2_ atmosphere at a scan rate of 10 K·min^–1^. Analysis of the proportion of metals in the samples was performed on a Philips XL30 ESEM scanning microscope equipped with an EDAX microprobe (SCSIE, Universitat de València).

## Results and Discussion

### Synthesis and Characterization

Crystalline europium(III) coordination polymers **1** and **2** were prepared by the reaction of Eu(NO_3_)_3_·6H_2_O with the appropriate picolinic acid under hydrothermal conditions. The addition of an acid modulator (nitric acid or acetic acid) was necessary in order to obtain single crystals suitable for structural characterization. Both compounds could also be obtained from the corresponding diesters in similar conditions.

The phase purity of the bulk materials was confirmed by PXRD measurements (Figure S1). The experimental diffractograms recorded at room temperature for compounds **1** and **2** compare well with the simulated patterns obtained from single-crystal data at 120 K.

Thermogravimetric analyses (TGA) of **1** and **2** were performed under a nitrogen atmosphere (Figure S2). For **1**, the TGA curve shows three separated steps of weight loss. The first one takes place between 416 and 435 K and is ascribed to the release of two coordinated water molecules (calcd.: 4.71%; found: 5.27%). The second and third steps occur above 540 K and are attributed to the decomposition of dimethylammonium cations and **L**_**1**_^**2–**^ ligands. These results are consistent with the formulation deduced from single-crystal X-ray diffraction measurements that show the presence of two water molecules per lanthanoid ion. For **2**, the TGA plot exhibits also a weight loss in multiple steps. Between RT and 390 K, a first weight loss associated with the release of water present in the voids of the structure takes place. A second step ascribed to the loss of acetic acid is detected between 440 and 580 K. The total weight loss for these first two steps is 9.26% (calcd.: 9.65%). Then, above 620 K decomposition of dimethylammonium cations and **L**_**2**_^**2–**^ ligands occurs. These observations are consistent with the release of two different types of solvent molecules and are in agreement with the formulation deduced from single-crystal X-ray diffraction. Clearly, **1** shows a higher dehydration temperature, indicating that water is more tightly bound to the anionic lattice as compared to **2**. This is expected for the presence of H_2_O molecules coordinating to the Eu^3+^ cation in **1**.

## Structural Properties

Single-crystal X-ray analysis reveals that **1** (Table 1) crystallizes in the monoclinic space group *I*2/*a*. Its asymmetric unit contains half Eu^3+^ cation, one **L**_**1**_^2–^ dianion, one coordinated water molecule, and half dimethylammonium cation ([(CH_3_)_2_NH_2_]^+^). This cation is required in order to balance the negative charge of the host framework and originates from the hydrolysis of dimethylformamide (DMF) solvent molecules.^52,53^

**Table 1:** Crystallographic Data

	**1**	**2**
chemical formula	C_32_H_26_EuN_3_O_10_	C_32_H_26_EuN_5_O_11_
*a* (Å)	11.97630(10)	19.6440(2)
*b* (Å)	10.67410(10)	12.35500(10)
*c* (Å)	24.7299(3)	13.21490(10)
α (deg)	90.00	90.00
β (deg)	98.0830(10)	90.00
γ (deg)	90.00	90.00
*V* (Å^3^)	3129.97(6)	3207.28(5)
*T* (K)	120.2(3)	119.7(8)
*Z*	4	4
*M*_*r*_ (g/mol)	764.52	808.54
crystal system	monoclinic	orthorhombic
space group	*I*2/*a* (No. 15)	*Pna*2_1_ (No. 33)
crystal dimensions (mm)	0.224 × 0.151 × 0.104	0.124 × 0.105 × 0.073
μ (Mo Kα) (mm^–1^)	2.066	2.025
λ (Å)	0.71073	0.71073
density (Mg/m^3^)	1.622	1.674
index ranges for *h*, *k*, *l*	–16/16, – 14/14, – 34/34	–25/24, – 16/16, – 16/17
θ range (deg)	3.189 to 29.753	3.298 to 28.107
goodness-of-fit on *F*^2^	1.153	1.082
reflns collected	37152	118251
independent reflns (*R*_int_)	4273 (0.0249)	7383 (0.0975)
data/restraints/parameters	4273/0/218	7383/33/453
*R*1, *wR*2 [*I* > 2σ(*I*)]^a^	0.0145, 0.0355	0.0389, 0.0765
*R*1, *wR*2 (all data)^a^	0.0158, 0.0363	0.0627, 0.0883
absolute structure parameter	n/a	–0.039(6)

a *R*1 = ∑(|*F*_0_| – |*F*_c_|)/∑|*F*_0_|; *wR*2 = [∑[*w*(*F*_0_^2^ – *F*_c_^2^)^2^]/ ∑[*wF*_o_^4^]]^1/2^.

**Figure 1:**
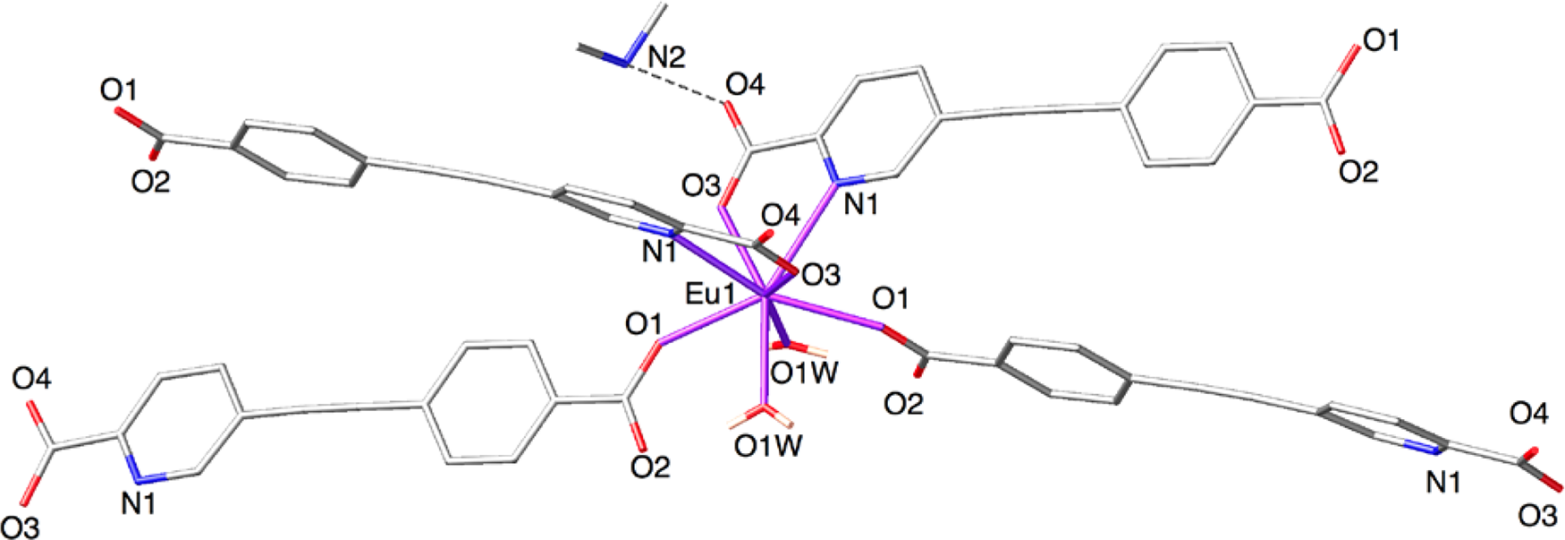
Stick plot of the Eu^3+^ coordination environment in **1**.

The Eu^3+^ cation sits on a two-fold axis and displays an eight-coordinated distorted square antiprism geometric configuration (Figure 1). It is coordinated by two chelating picolinate subunits (Eu1–O3: 2.4184(9) Å; Eu1–N1: 2.5918(11) Å), two monodentate benzoate groups (Eu1–O1: 2.3512(9) Å), and two water molecules (Eu1–O1W: 2.3799(10) Å). Eu–O bond distances are lower than the Eu–N distance, in agreement with previously reported values for Ln(III) complexes based on pyridyl-carboxylate ligands.^54^

Continuous shape measure (CShM) calculations using SHAPE 2.1 confirm that the coordination geometry is a distorted square antiprism (*D*_4*d*_, with a minimum CShM value of 9.458, Table S3).^55−58^

The ditopic ligand **L**_**1**_^2–^ bridges two Eu^3+^ cations through the chelating picolinate group and the benzoate anion acting in a monodentate mode. This connectivity results in the formation of a two-dimensional (2D) rhombus grid layer that presents channels (6.685 × 24.582 Å) along the *a* axis (Figure S3). These voids are occupied by dimethylammonium cations (one cation per channel) thanks to the establishment of hydrogen bonds with noncoordinated picolinate oxygen atoms (N2···O4: 2.7254(10) Å).

**Figure 2:**
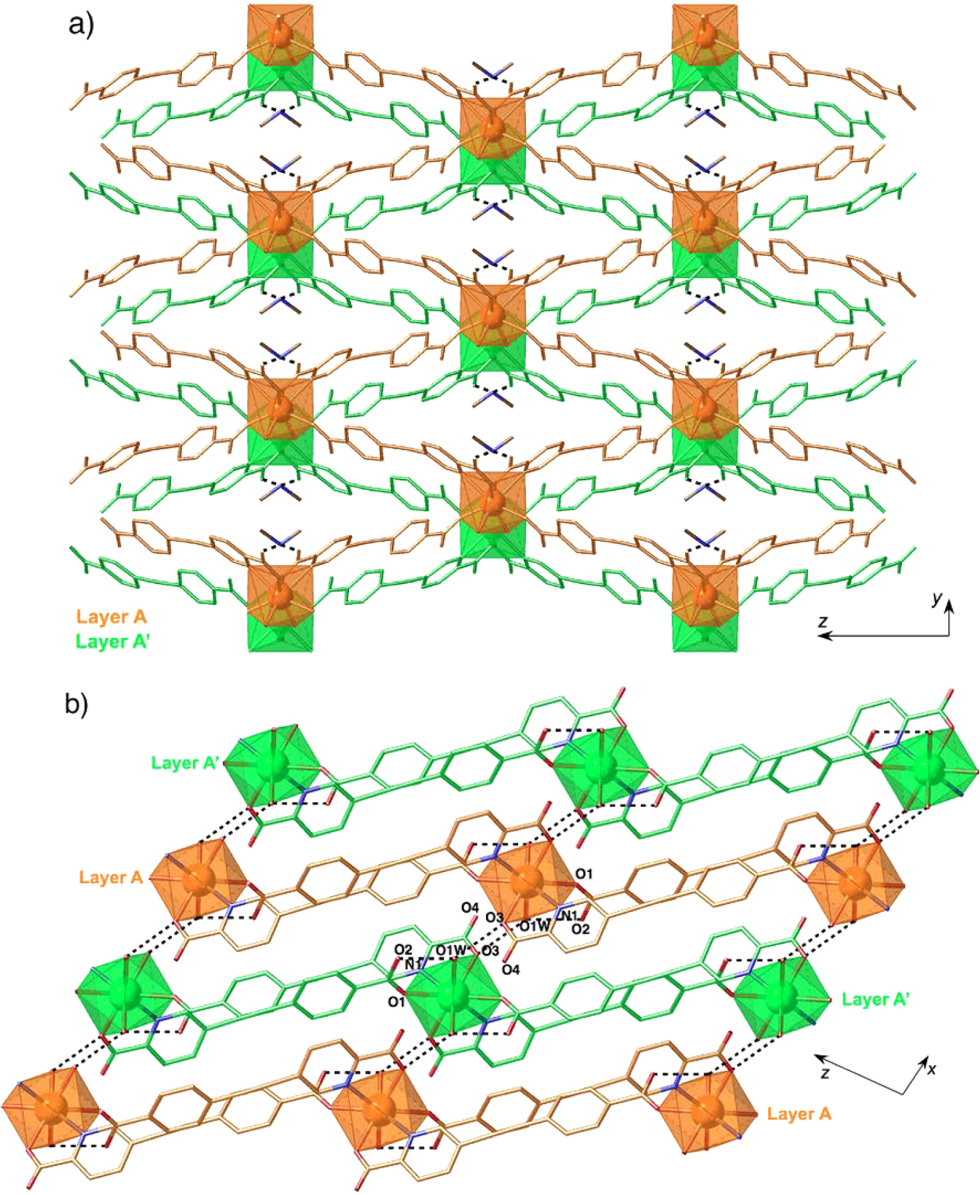
Projection of the crystal structure of **1** onto the *yz* (a) and *xz* (b) planes showing the layered AA′AA′ arrangement (each layer is depicted in a different color). H atoms have been omitted for clarity.

Additional hydrogen bonds between coordinated water molecules and carboxylate oxygen atoms (O1W···O3: 2.7677(14) Å, O1W···O2: 2.6092(14)) are established. The O1W···O2 interaction is a very strong *intramolecular* H-bond between water molecules and benzoate anions coordinating to the same Eu^3+^ cation, whereas the weaker *intermolecular* H-bond interaction involving the picolinate moiety O1W···O3 bridges complexes of consecutive layers, leading to a 3D doubly H-bonded network with an AA′AA′ arrangement (Figure 2a). In addition, π–π stacking interactions are observed between pyridine and benzene aromatic rings of adjacent layers (Figure 2b).

The anion exhibits an almost planar conformation, with a small dihedral angle between the pyridine and benzene rings (12.2(4)°) that allows coordination to two different metal cations. In addition, the carboxylate group involved in chelation presents a low torsion angle of 6.0(4)°, and the geometry of the triple bond is only slightly distorted (−C–C–C– bond angles of 178.0(4)° and 177.6(4)°, in comparison with the ideal value of 180°).

It is interesting to compare **1** with [(CH_3_)_2_NH_2_][Ln(H_2_O)_2_(CPA)_2_], a related family of lanthanoid complexes based on 5-(4-carboxyphenyl)picolinate dianions (CPA^2–^).^54^ Although the crystal structure of these compounds was reported in the *C*2/*c* space group, this could be converted to *I*2/*a* by the choice of a conventional unit cell^59^ with parameters similar to **1**. The lanthanoid coordination sphere is analogous to that described for **1**, and CPA^2–^ ligands adopt the same coordination mode to form an equivalent 2D rhombus grid layer. Even more, these layers are assembled into 3D networks via similar H-bonding and π–π stacking interactions. As expected, the size of the channels in **1** along its longest direction is notably higher in comparison with this family of compounds (7.278 Å × 19.161 Å for the Eu complex), due to the presence of the ethynylene spacer. However, when countercations are removed, the “SQUEEZE” option of PLATON^60^ indicates an empty volume of the anionic framework of 558.8 Å^3^ per unit cell for [(CH_3_)_2_NH_2_][Eu(H_2_O)_2_(CPA)_2_], corresponding to 20% of the total crystal volume. In compound **1**, a significantly lower value of 442.4 Å^3^, corresponding to a percent volume of 14%, is obtained indicating a more compact structure despite the presence of two additional carbon atoms in the ligand.

**Figure 3:**
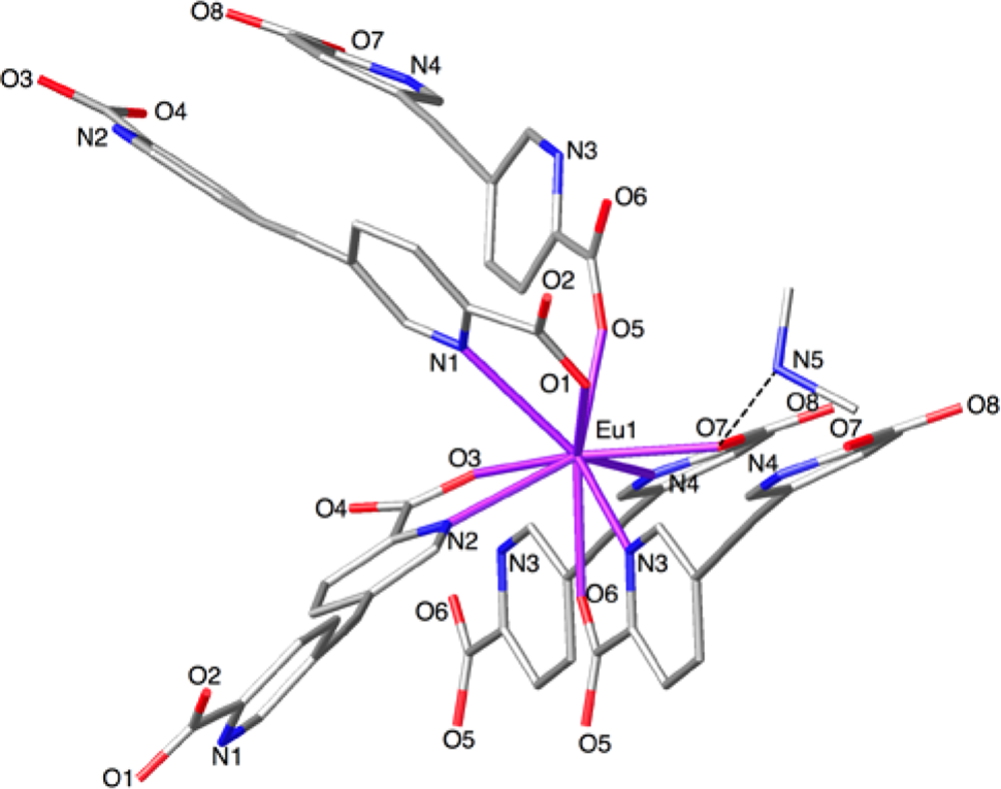
Stick plot of the Eu^3+^ coordination environment in **2**.

Compound **2** crystallizes in the orthorhombic *Pna*2_1_ space group (Table 1), and its asymmetric unit contains an Eu^3+^ cation, two crystallographically independent **L**_**2**_^2–^ anions, one dimethylammonium cation, and two crystallization solvent molecules (water and acetic acid). The Eu^3+^ cation is located in a general position and exhibits a nine-coordinated tricapped trigonal prismatic (TTP) structure as determined by continuous shape measure (CShM) calculations (Table S4 presents the CShM values for all the possible nine-coordinated-polyhedra, with a minimum CShM value of 3.882 for a tricapped trigonal prism.) Figure 3 shows the coordination sphere of the lanthanoid complex, with four picolinate subunits coordinating in a bidentate fashion, the ninth position being occupied by a bridging carboxylate oxygen atom (Eu1–O5: 2.363(6) Å). As in compound **1**, Eu–O bond distances (mean value: 2.376(6) Å) are shorter than Eu–N (mean value: 2.650(7) Å). Thus, the nitrogen atoms N2, N3, and N4 define the capping positions of the tricapped trigonal prism, the two triangular faces being defined by O3O6N2 and O1O5O7 atoms, respectively. The angle between the trigonal faces is 175.50°, close to the parallel arrangement expected for a TTP structure.

**Figure 4:**
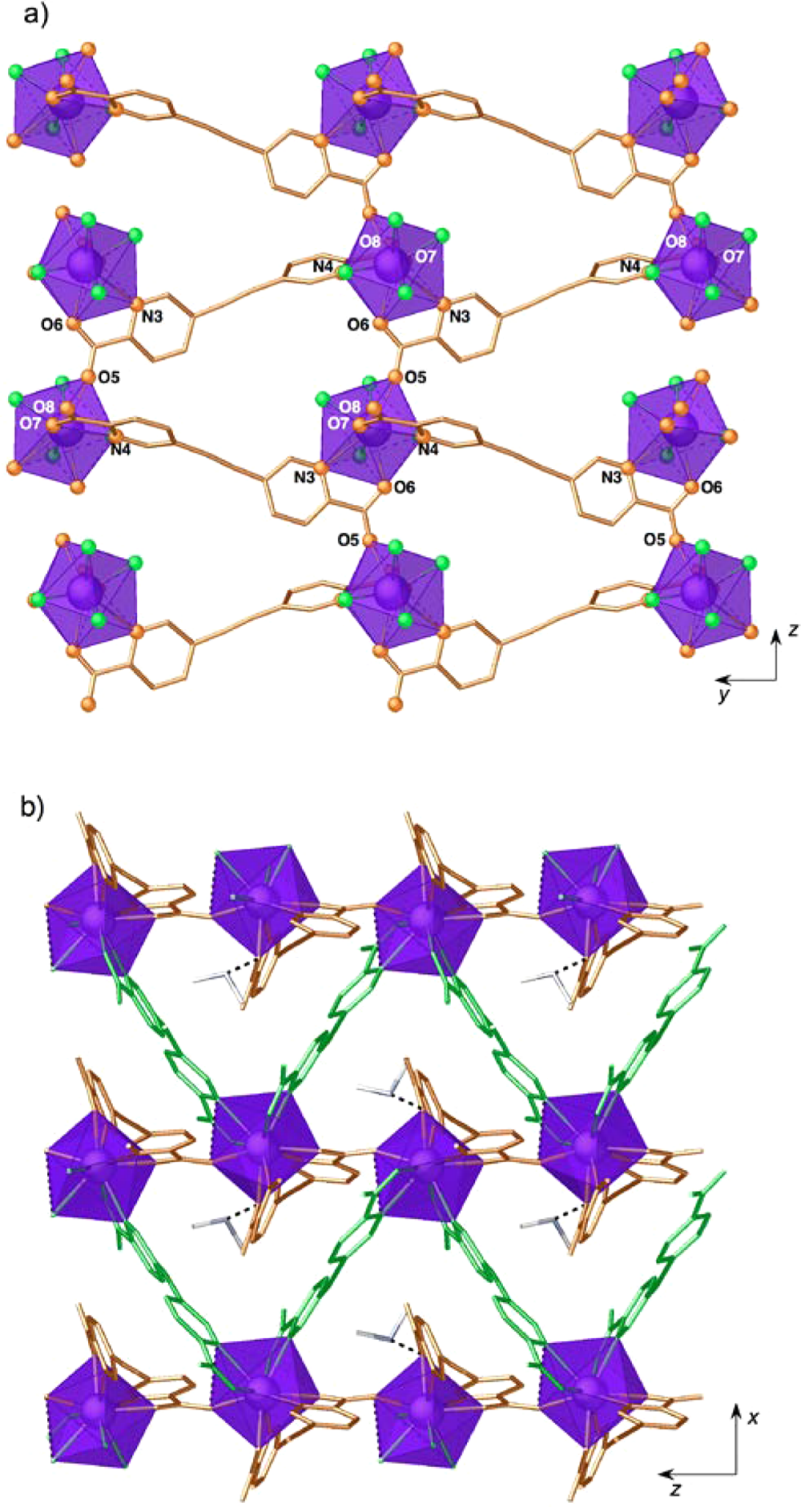
(a) View of the crystal structure of **2** along the *x* direction showing the layers defined by dianion ^**A**^**L**_**2**_^2–^. (b) Projection of the crystal structure of **2** onto the *xz* plane, showing the zigzag chains defined by dianion ^**B**^**L**_**2**_^2–^ and the presence of triangular voids in the 3D structure. H atoms have been omitted for clarity.

There are two independent bispicolinate **L**_**2**_^2–^ ligands (^**A**^**L**_**2**_^2–^ and ^**B**^**L**_**2**_^2–^) that adopt different coordination modes. Dianion ^**A**^**L**_**2**_^2–^ employs the two picolinate chelating subunits (N3O6 and N4O7) in coordination: one of them (N3O6) acts as a tridentate ligand in a (N,O);O mode, connecting two lanthanoid metal ions by *anti*, *anti*-carboxylate (O5O6) bridges. This yields zigzag chains running parallel to the *c* axis, with a relatively short intrachain distance between adjacent Eu^3+^ cations of 6.6891(7) Å (Figure S4). The other picolinate subunit (N4O7) coordinates in a terminal chelating manner, bridging neighboring chains related by a translation along the *b* axis. Thus, this first independent ligand defines layers that lie parallel to the *yz* plane (Figure 4a). The layers are further connected by the second dianion ^**B**^**L**_**2**_^2–^ acting as a simple bisbidentate ligand, with the two picolinate anions (N1O1 and N2O3) being bound in a terminal chelating manner to two Eu^3+^ cations separated by a long metal–metal distance of 12.8369(6) Å.

This results in a 3D anionic lattice showing triangular cavities (Figure 4b). Charge compensation is provided by dimethylammonium cations sitting within the cavities of the structure and interacting by hydrogen bonding with a coordinated picolinate oxygen atom (N5···O7:2.742(11) Å) and a water molecule (N5···O1W: 2.713(15) Å). Instead, the acetic acid molecule forms a short strong H-bond with a noncoordinated oxygen atom (O9···O4: 2.549(12) Å).

The two independent ligands adopt markedly different conformations: in ^**A**^**L**_**2**_^2–^, the two pyridine rings are almost perpendicular to each other (the dihedral angle between their mean planes is 100.2(9)°), whereas ^**B**^**L**_**2**_^2–^ shows a more planar *transoid* conformation, with a smaller dihedral angle of 27.2(9)°. In turn, the distortion of the triple bond is higher for ^**B**^**L**_**2**_^2–^ (−C–C–C– bond angles of 175.5(9)° and 171.5(9)°) than for ^**A**^**L**_**2**_^2–^ (−C–C–C– bond angles of 177.5(9)° and 179.7(9)°). As expected, the picolinate subunits, involved in chelation, are nearly planar, with the highest deviation from planarity being observed for the bridging tridentate picolinate anion (torsion angle between carboxylate and pyridine moieties of 12.3(9)°).

### Photoluminescent Properties

The electronic absorption spectra of free ligands H_2_**L**_**1**_ and H_2_**L**_**2**_ and their Eu^3+^ complexes were measured in the solid state at room temperature (Figure S5). The UV absorption spectra of both complexes show an intense absorption band at about 250–255 nm and a second absorption band at about 331–334 nm with the same intensity. These features are almost identical to those observed for the free ligands and are attributed to π → π* and *n* → π* electronic transitions of the pyridine and benzoate rings. The similarity observed between these patterns indicates that absorption takes place on the ligand rather than directly at the metal center.

**Figure 5:**
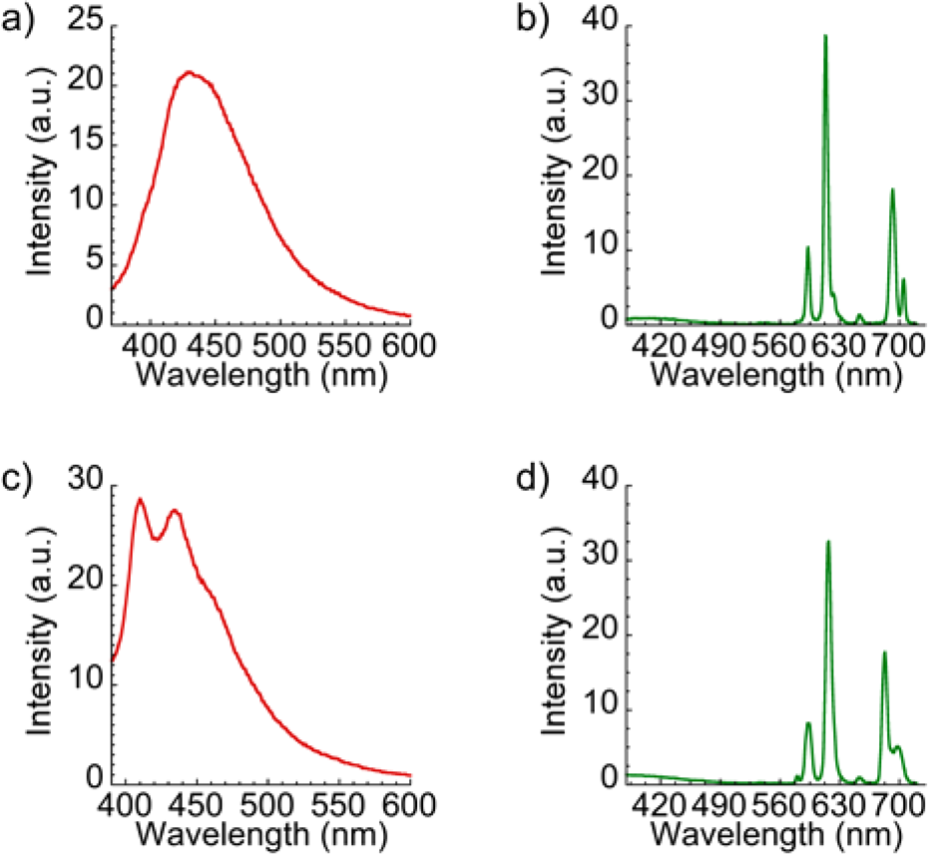
Emission spectra of ligands H_2_**L**_**1**_ (a, λ_exc_ = 350 nm), H_2_**L**_**2**_ (c, λ_exc_ = 356 nm), and Eu^3+^ compounds **1** (b, λ_exc_ = 344 nm) and **2** (d, λ_exc_ = 340 nm).

The photoluminescence properties of suspensions of compounds [(CH_3_)_2_NH_2_][Eu(H_2_O)_2_(**L**_**1**_)_2_] (**1**) and [(CH_3_)_2_NH_2_][Eu(**L**_**2**_)_2_]·H_2_O·CH_3_COOH (**2**) and ligands H_2_**L**_**1**_ and H_2_**L**_**2**_ were investigated in water at room temperature (Figure 5). After UV excitation around 350 nm, the free ligands exhibit an intense emission between 410 and 450 nm, which is attributed to π* → π and π* → *n* electronic transitions. Under excitation of complexes **1** and **2** in similar conditions, the typical red emission of the Eu^3+^ ion is observed, with emission peaks attributed to transitions between ^5^*D*_0_ and ^7^*F*_*J*_ levels (*J* = 0, 1, 2, 3, 4) (Table 2).^61^

**Table 2:** Luminescence Data for Complexes **1** and **2**^a^

**1** λ_em_ (nm)	**2** λ_em_ (nm)	assignment
578	580	^5^*D*_0_ → ^7^*F*_0_
593	592	^5^*D*_0_ → ^7^*F*_1_
614	615	^5^*D*_0_ → ^7^*F*_2_
653	653	^5^*D*_0_ → ^7^*F*_3_
705	690–696	^5^*D*_0_ → ^7^*F*_4_

a Peaks at 693 and 680 nm are attributed to the second harmonic of the laser for complexes **1** and **2**, respectively.

In both complexes, the emission spectrum is dominated by the most intense band at 615 nm ascribed to ^5^*D*_0_ → ^7^*F*_2_ transition, which is responsible for the strong red emission. In fact, the intensity ratio of the ^5^*D*_0_ → ^7^*F*_2_ transition and the ^5^*D*_0_ → ^7^*F*_1_ transition gives an indication about the symmetry of the first coordination environment of the Eu(III) ions and has a value of 3.6 for **1** and 3.8 for **2**. These values, together with the very low intensity of the ^5^*D*_0_ → ^7^*F*_0_ transition band, suggest a non-centrosymmetric environment for the metal center.^61^ The values of this ratio are very similar to those obtained for previous europium(III) complexes. For instance, [EuL(HL)H_2_O]·6H_2_O (being H_2_L bis(5-(pyridine-2-yl)-1,2,4-triazol-3- yl)methane ligand) presents a ratio of 3.4.^62^

It is worth noting that there is a negligible contribution below 500 nm coming from the ligand in the emission spectrum of complexes **1** and **2**. Therefore, both ligands have proven to be suitable for absorbing light efficiently and transferring this energy to the europium cations with high efficiency. In fact, the excitation spectra of both complexes (Figure S6) monitored within the Eu^3+^ most intense transition (^5^*D*_0_ → ^7^*F*_2_) are dominated by a broad band from 310 to 390 nm and from 220 to 375 nm, respectively, corresponding to intraligand transitions. There is also a weak signal at 395 nm attributed to a transition between the ^7^*F*_0_ and excited states of Eu^3+^. The very low relative intensity of this line compared with that of the ligand-based broad band suggests a more efficient ligand-sensitization process than direct intra-4f^6^ excitation.

**Figure 6:**
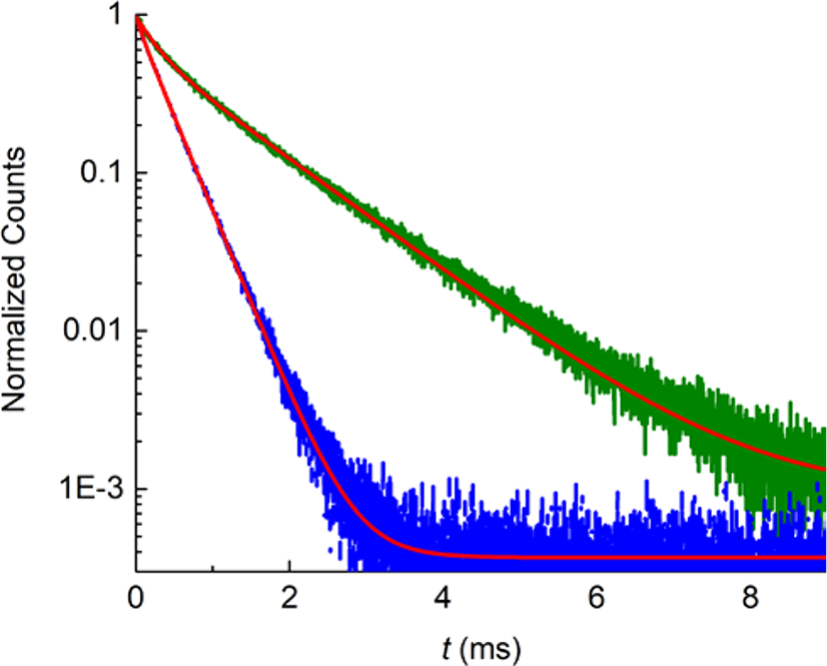
Luminescence decay curves of **1** (blue) and **2** (green) (λ_em_ = 614 nm). Best-fit data to a biexponential function are shown in red. The samples were excited with a 375 nm laser at a frequency of 20 Hz.

Luminescence decay curves of **1** and **2** in the solid state were measured at room temperature by monitoring the time dependence of the strongest ^5^*D*_0_ → ^7^*F*_2_ emission (Figure 6). In both cases, the decrease in the number of excited fluorophores following optical excitation with a short light pulse was successfully fitted using a biexponential function (eq 1), suggesting the existence of two different pathways for luminescence decay:
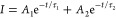
1where τ_*i*_ is the lifetime and *A*_*i*_ is the pre-exponential factor that allows quantitative assignment of the relative contribution of each component. The best-fit data for compound **1** yielded τ_1_ = 84 μs and τ_2_ = 367 μs, with *A*_1_ = 0.0953 and *A*_2_ = 0.8848, and a χ^2^ value of 0.999. An average lifetime τ_av_ = 360 μs was calculated by the following expression:^63^
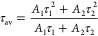
2

Instead, for compound **2**, values of τ_1_ = 274 μs and τ_2_ = 1222 μs, with *A*_1_ = 0.3514 and *A*_2_ = 0.6258 (χ^2^ = 0.999), were obtained. This corresponds to an average lifetime τ_av_ = 1116 μs. Luminescence lifetimes on the micro- to millisecond time scale are typical for f–f transitions that involve long-lived excited states, and the values obtained for **1** and **2** are within this range (Table S5).^64−68^ However, **2** shows clearly higher lifetime values than **1**. This can be explained taking into account that **1** presents two H_2_O molecules in the coordination sphere of the Eu^3+^ cation. It is well-known that nonradiative decay of the excited states of Eu^3+^ takes place via vibronic coupling with the vibrational modes of O–H bonds of coordinated water molecules, leading to a significant reduction of excited state lifetimes.^69^ This is why the luminescence of lanthanoid-based complexes is markedly influenced by the denticity of the ligand, polydentate ligands being useful for increasing the stability of the complexes and allowing the metal center to be protected from solvent molecules.

Photoluminescence quantum yields (PLQY) were measured by placing the samples into an integrating sphere. Values of 23% and 29% were obtained for complexes **1** and **2**, respectively. These values are typical for Eu^3+^ coordination polymers (Table S5). The slightly higher value obtained for **2** parallels the lifetime measurements and is due to the lack of coordinating water molecules. This behavior has been well documented for many hydrated europium diketonate complexes.^70^ Nonetheless, a recent report on [EuKL_4_(H_2_O)_2_]·H_2_O (being HL = 7-chloro-1-cyclopropyl-6-fluoro-4-oxo-1,4-dihydroquinoline-3-carboxylic acid) presents an ultrahigh quantum yield value of 92%, which is much higher than for most lanthanoid complexes.^71^

### Luminescence Sensing of Solvent Molecules

**Figure 7:**
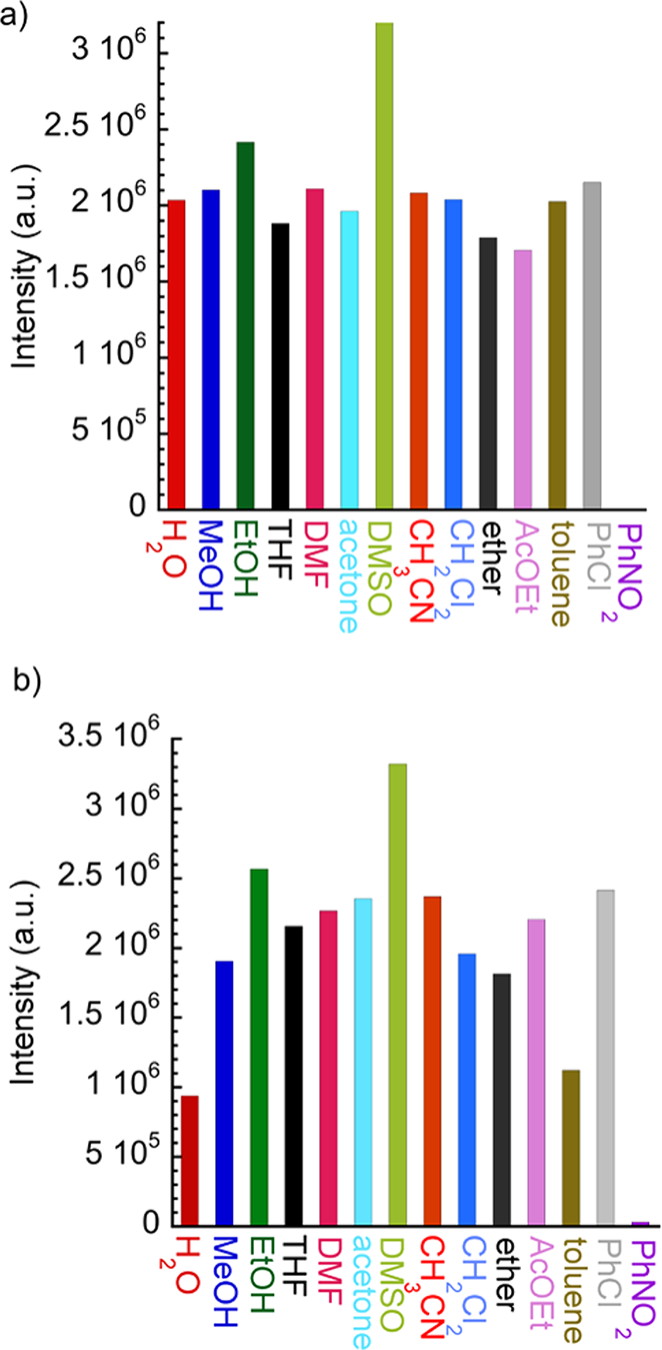
Bar plots of emissive intensities (peak height at λ = 614 nm) for **1** (a, λ_exc_ = 344 nm) and **2** (b, λ_exc_ = 340 nm) dispersed in different solvents.

The luminescence sensing response of both europium derivatives for different solvent molecules was studied at room temperature. The as-synthesized crystals (1 mg) were ground and suspended in different solvents (1 mL), and the corresponding emission spectra were recorded (Figure S7). The intensity of the strongest peak that corresponds to the ^5^*D*_0_ → ^7^*F*_2_ transition was taken for comparison. The luminescence signal of **1** (Figure 7a) was enhanced by solvents like MeOH, EtOH, CH_3_CN, DMF, and DMSO, presenting in the last case a huge increase of 72% (*I*(DMSO)/*I*(H_2_O) ≈ 1.72). For other solvent molecules, the emission intensity was slightly reduced. Remarkably, nitrobenzene (PhNO_2_) could effectively quench the luminescence of **1**. For **2**, a general enhancement of the luminescence signal was observed when a solvent different to water was used, except for PhNO_2_, that exhibited an almost complete luminescence quenching (Figure 7b). Again, a remarkable luminescence increase (*I*(DMSO)/*I*(H_2_O) ≈ 3.6) was observed in DMSO suspensions.

In order to investigate these luminescence enhancement and quenching effects (Figure S8), powder X-ray diffraction (PXRD) measurements of both Eu^3+^ coordination polymers were carried out after the corresponding solid samples were suspended in DMSO and PhNO_2_. A perfect agreement between these diffractograms and those corresponding to the as-synthesized materials was obtained, indicating that their crystal structures are retained after interaction with these solvent molecules.

An increase of emission intensity after soaking in DMSO has been previously noted in Eu^3+^ complexes and ascribed to energy transfer from DMSO to the antenna ligand.^72^ However, in this case DMSO molecules do not absorb energy at the excitation wavelengths used (340 and 344 nm, Figure S9), discarding this hypothesis. Thus, a possible explanation for this fact relies on the exchange of inner-sphere coordinating water molecules or hydrogen-bonded water molecules by DMSO *on the surface of the particles*, which would preserve their crystal integrity. In this way, nonradiative decay of the excited states through vibronic coupling with the vibrational modes of O–H bonds would be reduced, with the corresponding enhancement in the europium emission.

Regarding nitrobenzene quenching, the disruption of the crystal structure can also be excluded since the diffraction patterns remain unaltered after dispersing the solids in this solvent. The absorption spectrum of PhNO_2_ was compared to the emission spectra of **1** and **2** (Figure S9). The lack of spectral overlap between absorption and emission patterns discards a resonance energy transfer process between Eu^3+^ ions and PhNO_2_. Nevertheless, there is a spectral overlap between the absorption spectrum of PhNO_2_ and the emission of ligands H_2_**L**_**1**_ and H_2_**L**_**2**_, indicating that a Förster resonance energy transfer (FRET) might be at the origin of the quenching process. However, since PhNO_2_ can absorb energy at the excitation wavelength used in these experiments (344 and 340 nm, respectively), a competitive absorption mechanism cannot be excluded. Energy competition between Eu complexes and nitroaromatic compounds has been already noted as a possible mechanism of luminescence quenching.^73,74^

**Figure 8:**
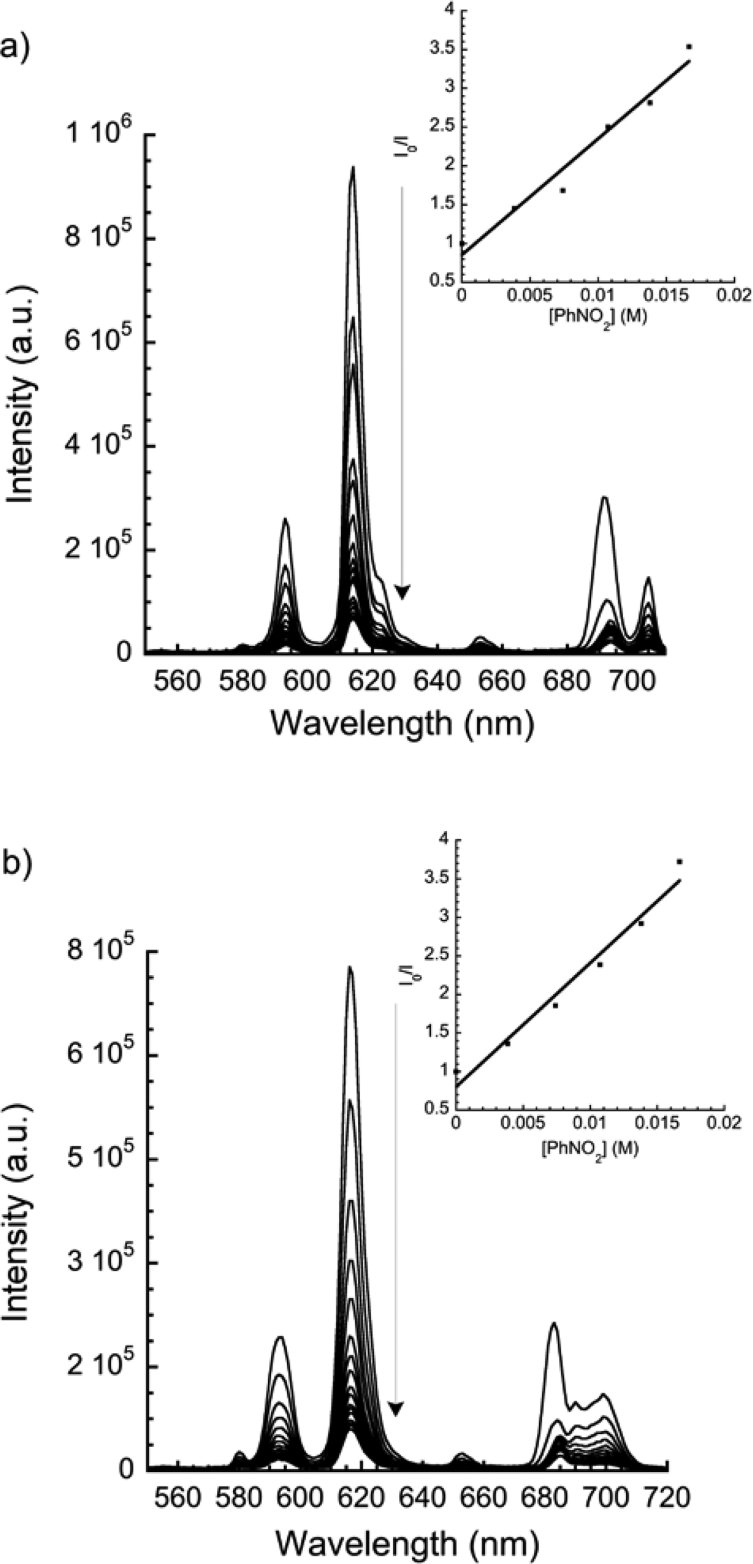
Emission spectra of **1** (a, λ_exc_ = 344 nm) and **2** (b, λ_exc_ = 340 nm) upon addition of different volumes of PhNO_2_ (0.1 M in EtOH). Insets show Stern–Volmer plots for **1** (a) and **2** (b) in the low [PhNO_2_] region, together with data fits to the Stern–Volmer equation.

The above results suggest that **1** and **2** could be used as specific luminescent sensors for nitroaromatic molecules. Thus, titration experiments with PhNO_2_ (0.1 M in EtOH) were undertaken on aqueous suspensions of **1** and **2**. Both complexes exhibited a very similar gradual decrease of the luminescence intensity as the concentration of PhNO_2_ was increased (Figure 8a,b). The luminescence intensity versus PhNO_2_ concentration plot was fitted by using the well-known Stern–Volmer equation (insets Figure 8a,b):^75,76^
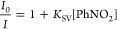
3Stern–Volmer plots display a linear variation at a low concentration of quencher, while they present a quadratic dependence at higher concentrations. This indicates the existence of two different relaxation mechanisms, static and dynamic quenching.^76^ The calculated Stern–Volmer quenching constants (*K*_SV_) were 1.50 × 10^2^ M^–1^ and 1.60 × 10^2^ M^–1^ for **1** and **2**, respectively, indicating that both compounds show a moderate nitrobenzene sensing ability in comparison to other Eu(III) luminescent sensors (Table S2). Moreover, the limit of PhNO_2_ detection (LOD) was calculated based on the following equation:
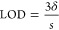
4where *s* is the slope of the plot of luminescence intensity against PhNO_2_ concentration in the linear region (Figure S10) and δ is the standard deviation for 10 repeated luminescence measurements of the blank solution. The results show detection limits of 2.05 × 10^–5^ M and 3.03 × 10^–5^ M for **1** and **2**, respectively. These values compare well with those reported for similar Eu(III) complexes (Table S2).

### Luminescence Sensing of Metal Ions

**Figure 9:**
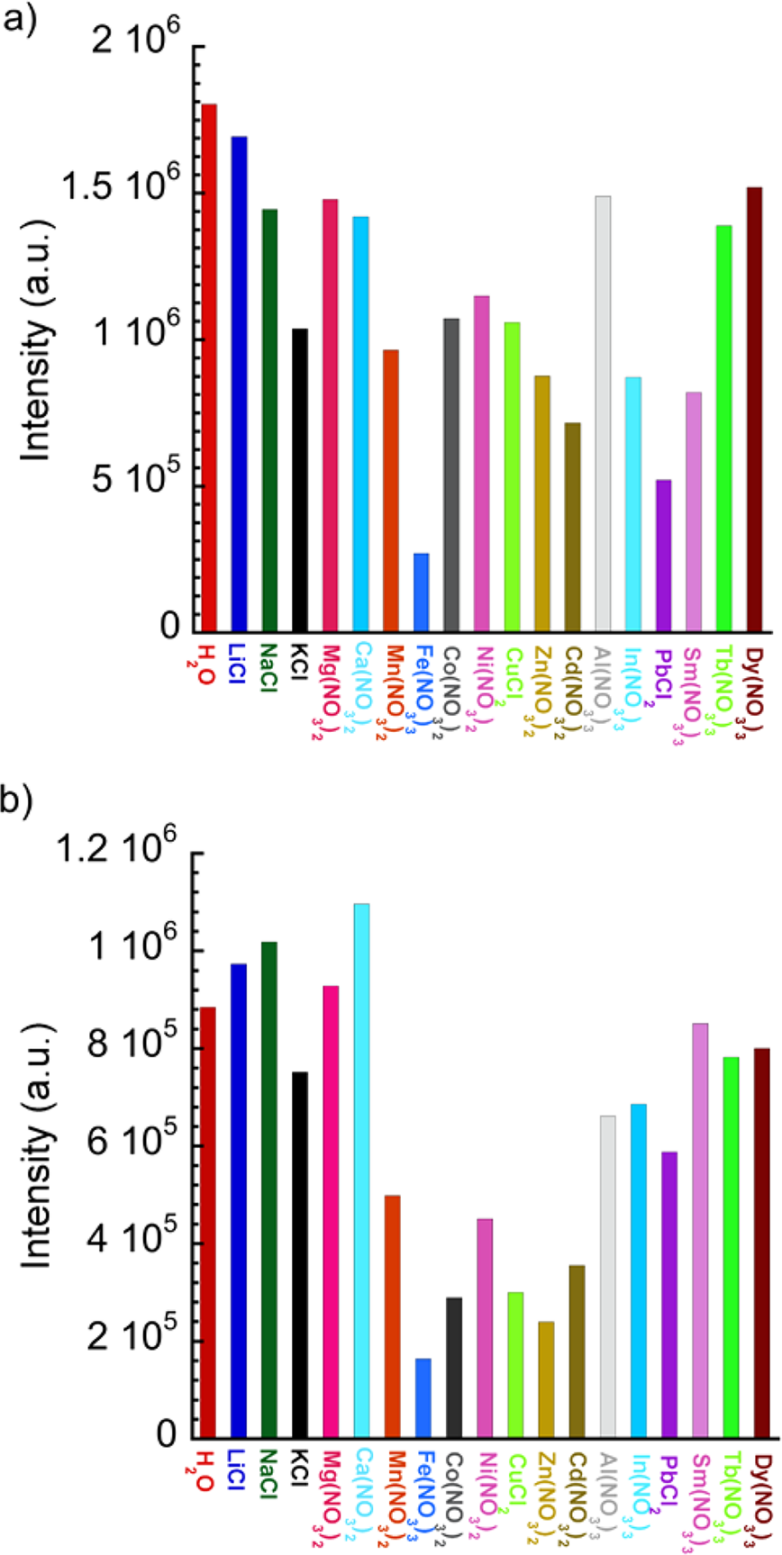
Bar plots of emissive intensities (corresponding to the ^5^*D*_0_ → ^7^*F*_2_ transition) for **1** (a, λ_exc_ = 344 nm) and **2** (b, λ_exc_ = 340 nm) in 0.01 M aqueous solutions of different metal ions.

Coordination polymers **1** and **2** (1 mg) were dispersed in 1 mL aqueous solutions containing 0.01 M of MCl_*n*_ or M(NO_3_)_*n*_ metal salts (M = Li^+^, Na^+^, K^+^, Mg^2+^, Ca^2+^, Mn^2+^, Fe^3+^, Co^2+^, Ni^2+^, Cu^2+^, Zn^2+^, Cd^2+^, Al^3+^, In^3+^, Pb^2+^, Sm^3+^, Tb^3+^, and Dy^3+^), and then the luminescence spectra were recorded (Figure S11). Again, in order to analyze the data, the strongest peak, corresponding to the ^5^*D*_0_ → ^7^*F*_2_ transition, was taken for comparison. For **1**, all metal ions exhibited luminescence quenching effects (Figure 9a). Noteworthy is the fact that Fe^3+^ and Pb^2+^ ions could almost completely quench its luminescence, Fe^3+^ being more effective than Pb^2+^ ions (up to 80% and 72%, respectively). On the other hand, metals like Li^+^, Na^+^, Mg^2+^, and Ca^2+^ increased the emission intensity of **2**, the remaining metal ions resulting in different degrees of luminescence quenching (Figure 9b). Fe^3+^, Cd^2+^, and Zn^2+^ metal ions stand out with quenching efficiencies of 82, 68, and 69%, respectively. These results are consistent with the fact that Pb^2+^ ions present higher affinity for oxygen donor ligands than for nitrogen-based ligands. That means that Pb^2+^ ions exhibit a greater competition for **L**_**1**_ ligand coordination with respect to **L**_**2**_, the former containing an additional carboxylate functional group. Ln^3+^, Mg^2+^, and Ca^2+^ cations present also higher affinity for oxygen donor ligands. Therefore, the decrease in the emission intensity is higher for **1** in these cases as well. Instead, transition metal cations like Co^2+^, Ni^2+^, Cu^2+^, Zn^2+^, and Cd^2+^ exhibit a higher affinity for nitrogen donor ligands, and this is in agreement with the stronger quenching effect observed for **2**. In general, **2**, containing the bis(picolinate) ligand, shows a better stability in these metal-sensing experiments than **1**.

PXRD measurements were conducted (Figure S12) in order to check the stability of the crystalline frameworks after interaction with Fe^3+^ and Pb^2+^ cations. PXRD patterns of **1** and **2** suspended in aqueous solutions of Fe^3+^ cations are consistent with the original frameworks, whereas the pattern of **1** suspended in an aqueous solution of Pb^2+^ cations is completely different. This indicates that collapse of the original network takes place, possibly resulting from the interaction between Pb^2+^ cations and the uncoordinated carboxylate groups in the channel. Energy-dispersive X-ray (EDX) microanalysis carried out on a scanning electron microscope (SEM) showed that this sample contains both Pb^2+^ and Eu^3+^ ions, with a Pb^2+^/Eu^3+^ ratio of 1.3. The same analysis for the compounds soaked in aqueous solutions of Fe^3+^ ions indicates that these cations are not present in the composition of the soaked materials. Therefore, the collapse of the framework of **1** induces the variation of the luminescence intensity after immersion in a Pb^2+^ aqueous solution, but this can be ruled out for Fe^3+^ sensing. In order to get more insight into the iron(III) quenching mechanism, electronic absorption spectra were recorded for aqueous solutions of the different metal ions and compared to the emission spectra of **1** and **2**, together with those of corresponding ligands (Figure S13). The lack of overlap between the absorption spectra of the different metallic ions and the emission spectra of the complexes discards a resonance energy transfer mechanism for luminescence quenching. Nonetheless, there is a considerable overlap between the absorption spectrum of Fe^3+^ ions and the emission spectra of ligands **H**_**2**_**L**_**1**_ and **H**_**2**_**L**_**2**_. Energy transfer from excited levels of the ligand to the Fe^3+^ ions could be a possible quenching mechanism. On the other hand, the wide absorption band of Fe^3+^ ions ranging from 430 to 260 nm covers the wavelength used for excitation of **1** and **2** (344 and 340 nm, respectively), while other metal ions such as Pb^2+^ have no absorption in this range. Therefore, competitive absorption by Fe^3+^ ions could also account for the quenching process.

**Figure 10:**
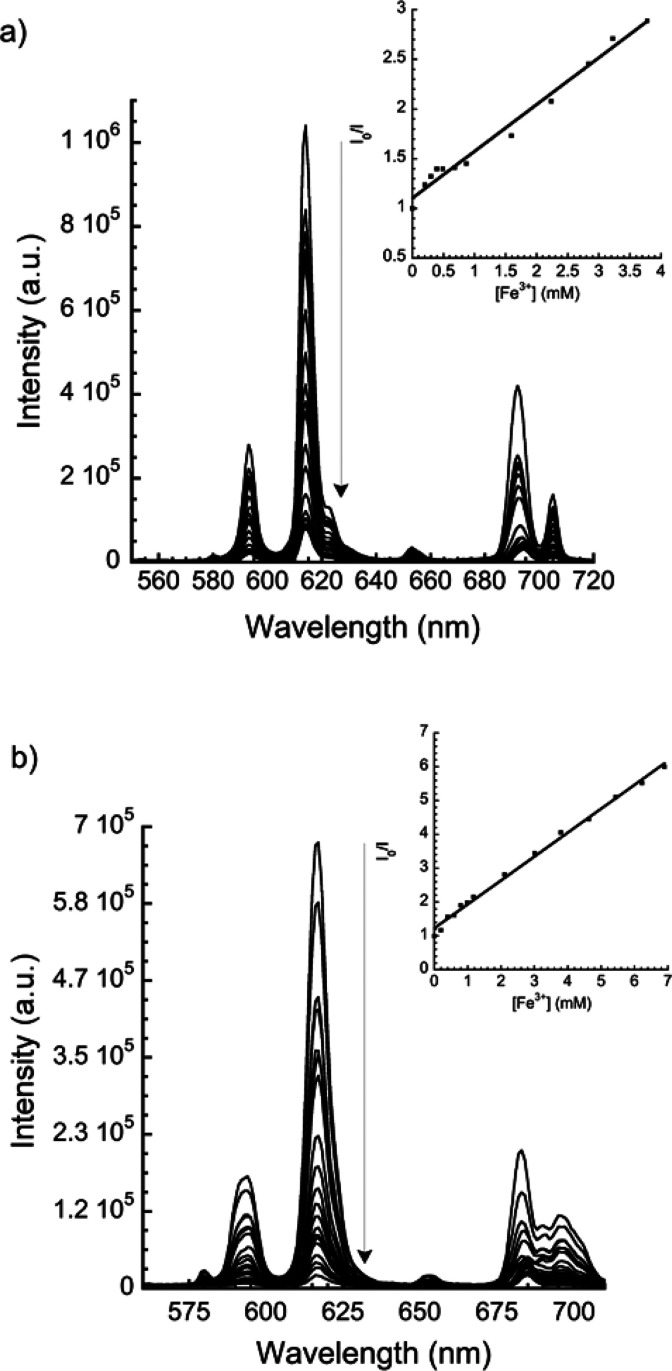
Emission spectra of **1** (a, λ_exc_ = 344 nm) and **2** (b, λ_exc_ = 340 nm) upon addition of different volumes of a Fe^3+^ aqueous solution (25 mM for **1** and 50 mM for **2**). Insets show the respective Stern–Volmer plots corresponding to the low [Fe^3+^] region, together with a data fit to the Stern–Volmer equation.

Selectivity and sensitivity of both complexes toward Fe^3+^ detection were also studied. First, competitive experiments containing Fe^3+^ and different metal ions showed that quenching of the emission is due only to the presence of the ferric species (Figure S14). A suspension of each complex (1 mg in 1 mL H_2_O) was then titrated with Fe^3+^ aqueous solutions of concentrations 25 mM and 50 mM, respectively, for **1** and **2**. The luminescence of the Eu^3+^ polymers was gradually quenched (Figure 10). Further, in order to discard dilution effects, the emission of both suspensions prepared in an equivalent volume of water was registered (Figure S15). There is a clear difference between the intensities recorded in the presence and in the absence of Fe^3+^ ions, verifying that a quenching process is present.

The quenching efficiency was quantitatively evaluated using the Stern–Volmer eq 3, in this case Fe^3+^ being the quencher species. The Stern–Volmer plots present again an upward bending at a high concentration of quencher, characteristic of the existence of static and dynamic quenching mechanisms.^76^ From the fit of the experimental data at low Fe^3+^ concentrations (from 0.19 to 3.77 mM and from 0 to 6.9 mM, respectively, for **1** and **2**) to the Stern–Volmer equation (insets of Figure 10), *K*_SV_ values of 4.71 × 10^2^ M^–1^ and 7.06 × 10^2^ M^–1^ are obtained for **1** and **2**, respectively. These values are comparable to those obtained by other previously reported Eu^3+^ coordination polymers (10^3^–10^4^ M^–1^, Table S1).^55^

Furthermore, the limit of detection (LOD) was calculated from 3δ/*s* (eq 4, where δ is the standard deviation of 10 consecutive blank measurements, and *s* is the slope of luminescence intensity vs [Fe^3+^] plot in the linear region), giving a value of 5.82 × 10^–6^ M and 3.16 × 10^–6^ M for **1** and **2**, respectively (Figure S16). Both values are very similar and within the range reported for other sensors based on Eu^3+^ coordination polymers (Table S1) but still far from the 10^–7^ M value obtained for [(CH_3_)_2_NH_2_][Eu(CPA)_2_(H_2_O)_2_] (CPA^2–^ = 5-(4-carboxyphenyl)picolinate dianion), one of the highest reported to date.^54^

**Figure 11:**
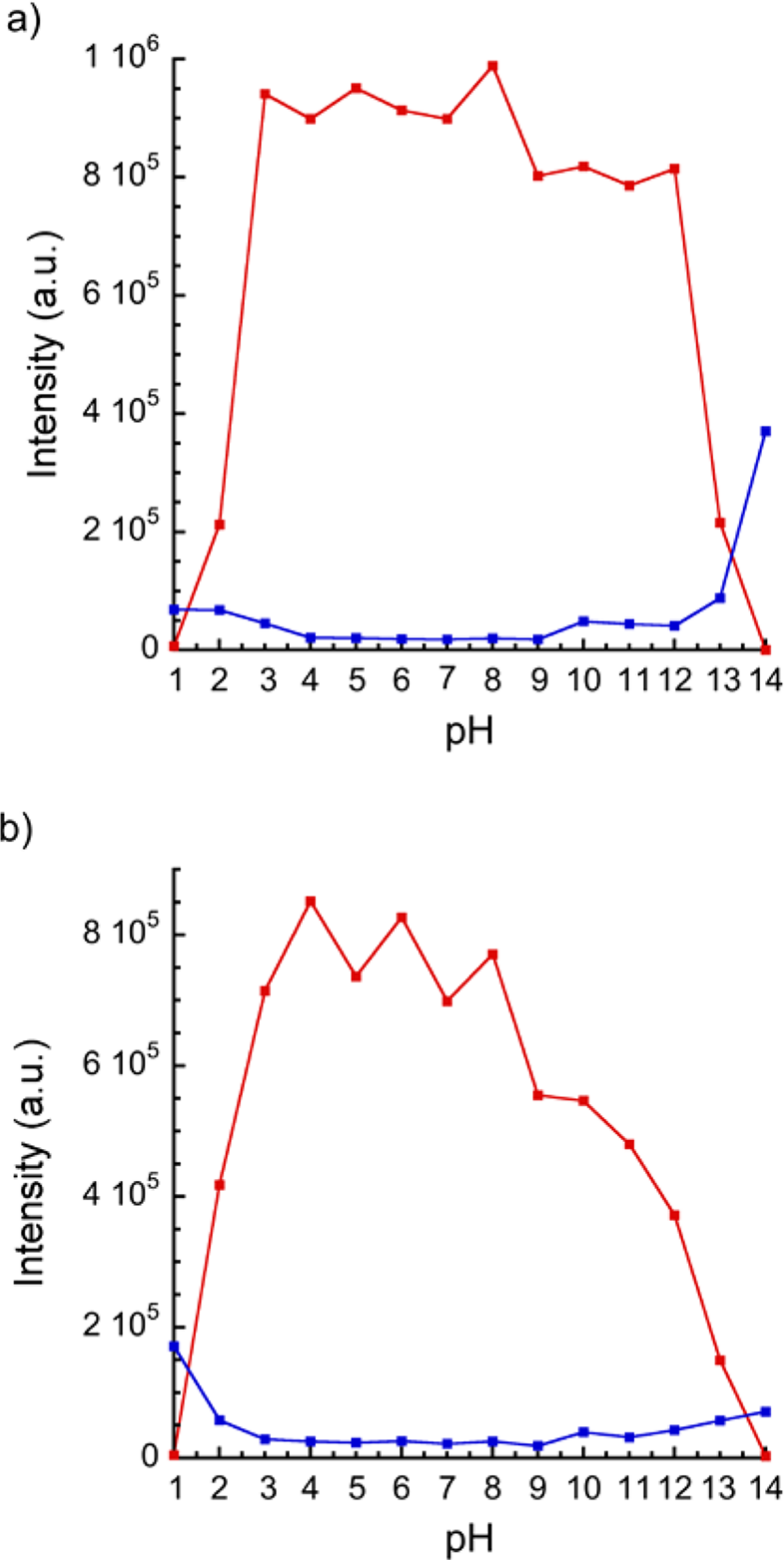
pH dependence of the emission intensity of **1** (a) and **2** (b) (red dots: Eu^3+^ emission at 614 nm; blue dots: ligand-based emission at 396–432 nm for **1** and 400–376 nm for **2**).

Luminescence studies as a function of pH were carried out for both complexes in water suspensions. The emission was registered at different pH values ranging from 1 to 14. The pH of each aqueous solution was adjusted by the addition of HCl or NaOH solutions. For **1**, it was found (Figure 11a) that Eu-based emission is almost constant in the pH range comprised between 3 and 12, whereas at extreme acid or basic pH values, there is a complete disappearance of the luminescence, together with the onset of a molecular fluorescence emission (λ_max_ = 396–432 nm), which is attributed to the decomposition of the complex and release of the free ligand in its protonated or fully deprotonated forms.^62^ The remarkable stability of **1** was demonstrated after soaking the coordination polymer in an aqueous solution at pH = 12 for 12 h (Figure S17). The powder X-ray diffractogram of the solid obtained was identical to that of the pristine material. This is in contrast with previous reports showing a lack of stability in most cases.^71^ There are only two previous reports showing a similar robustness in these conditions,^77,78^ but these compounds exhibit higher LOD in comparison with **1**. Instead, **2** displays the strong emissive properties in a shorter pH range (3 < pH < 8) (Figure 11b). Again, a molecular-based emission (at 400–376 nm) is observed at pH = 1, 13, and 14, which is ascribed to the release of free ligand at extreme pH values. PXRD experiments (Figures S18–S19) confirm the collapse of the crystal structure at pH = 1 and pH = 14 for both compounds. The lower stability of **2** outside the 3–8 pH range was also confirmed by the presence of minor diffraction peaks in the X-ray diffractogram.

## Conclusions

To summarize, two water-stable luminescent Eu^3+^ coordination polymers **1** and **2** have been synthesized from ethynylene-bridged ditopic picolinate ligands by the hydrothermal method. Suspensions of these compounds in water exhibited a strong Eu^3+^ emission under UV light irradiation, characteristic of effective ligand-to-metal energy transfer (antenna effect). Luminescence could be quenched by nitrobenzene, with limits of detection of 2.05 × 10^–5^ M and 3.03 × 10^–5^ for **1** and **2**, respectively. These coordination polymers were also able to act as luminescent sensors for Fe^3+^ cations, with limits of detection in the micromolar region (5.82 × 10^–6^ M and 3.16 × 10^–6^ for **1** and **2**, respectively). Noteworthy is the very high stability of these coordination polymers under extreme pH conditions.

## Supporting Information Available

Figures S1 (PXRD measurements), S2 (TGA), S3–S4 (structure plots), S5 (absorption spectra), S6 (excitation spectra), S7 (emission spectra in different solvents), S8 (PXRD in different solvents), S9 (spectral overlap with different solvents), S10 (luminescence vs [PhNO_2_] plot), S11 (emission spectra in the presence of different metal ions), S12 (PXRD in the presence of different metal ions), S13 (spectral overlap in the presence of different metal ions), S14 (selective Fe^3+^ sensing experiments), S15 (dilution experiments), S16 (luminescence vs [Fe^3+^] plot), S17 (stability of **1** at pH = 12), S18–S19 (PXRD of **1** and **2** at different pH values), and Tables S1–S2 (reported Stern–Volmer constants and LOD values for detection of Fe^3+^ cations and nitrobenzene, respectively), S3–S4 (continuous shape measures for **1** and **2**, respectively) and S5 (reported quantum yield and lifetime values for selected Eu^3+^ complexes) (PDF)

## Accession Codes

CCDC 2068840 and 2068875 contain the supplementary crystallographic data for this paper. These data can be obtained free of charge via www.ccdc.cam.ac.uk/data_request/cif, or by emailing data_request@ccdc.cam.ac.uk, or by contacting The Cambridge Crystallographic Data Centre, 12 Union Road, Cambridge CB2 1EZ, UK; fax: +44 1223 336033.
