# Supplementary material for: Robust Lanthanoid Picolinate-Based Coordination Polymers for Luminescence and Sensing Applications
Source: Inorg Chem. 2021 Jul 7;60(14):10572–84. doi: 10.1021/acs.inorgchem.1c01229 (PMC8454995; doi:10.1021/acs.inorgchem.1c01229)
Supplement: Supplementary file 1 — ic1c01229_si_001.pdf [file ic1c01229_si_001.pdf]

Supporting Information

Robust Lanthanide Picolinate-Based Coordination Polymers for Luminescence and Sensing Applications

*Verónica Jornet-Mollá, Chris Dreessen and Francisco M. Romero**

Instituto de Ciencia Molecular, Universitat de València, P. O. Box 22085, 46071 València, Spain; orcid.org/0000-0002-1936-0781; Email: fmrm@uv.es

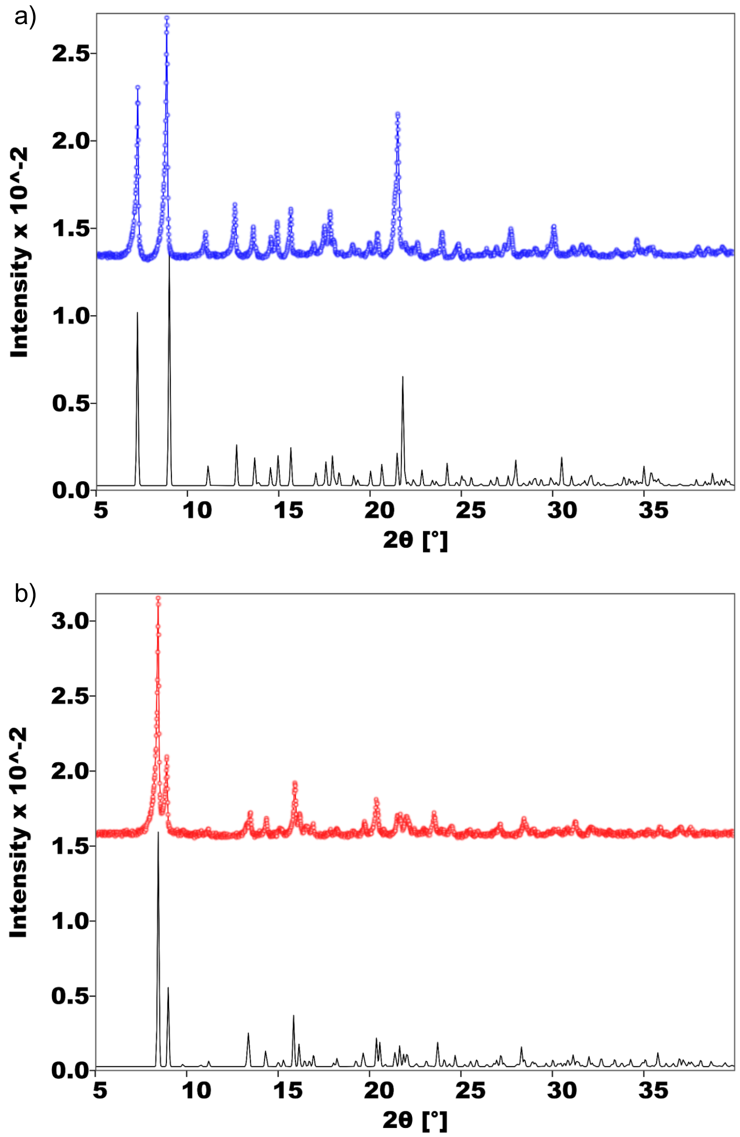

Figure S1. Comparison of the powder X-ray diffractograms of **1** (a, blue) and **2** (b, red) with the simulation obtained from the corresponding single crystal X-ray data using *CrystalDiffract* software (shown in black).

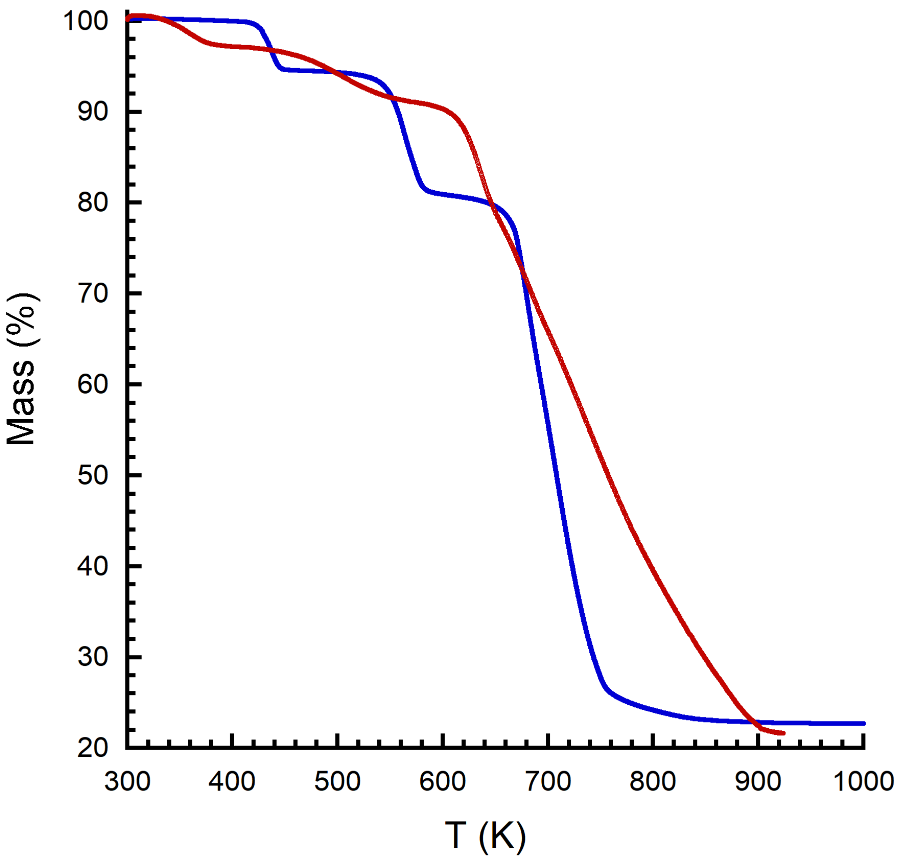

Figure S2. Thermogravimetric analysis of **1** (blue) and **2** (red).

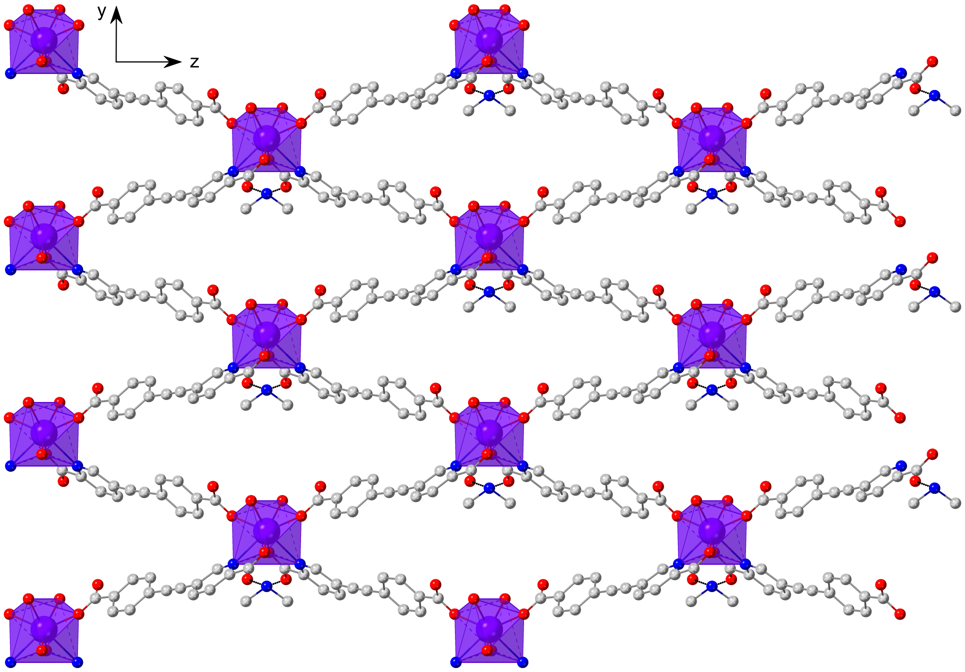

Figure S3. Representation of the layered structure of **1** in the *yz* plane.

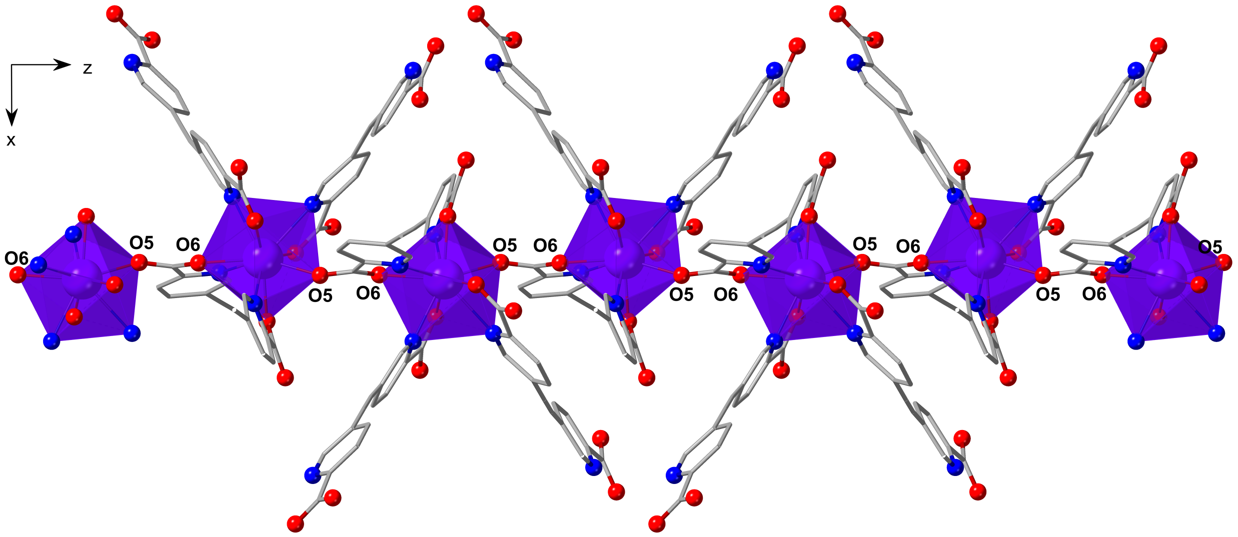

Figure S4. View of the crystal structure of **2** showing the carboxylate-bridged chain of Eu^{3+} cations running along the *z* direction. H atoms have been omitted for clarity.

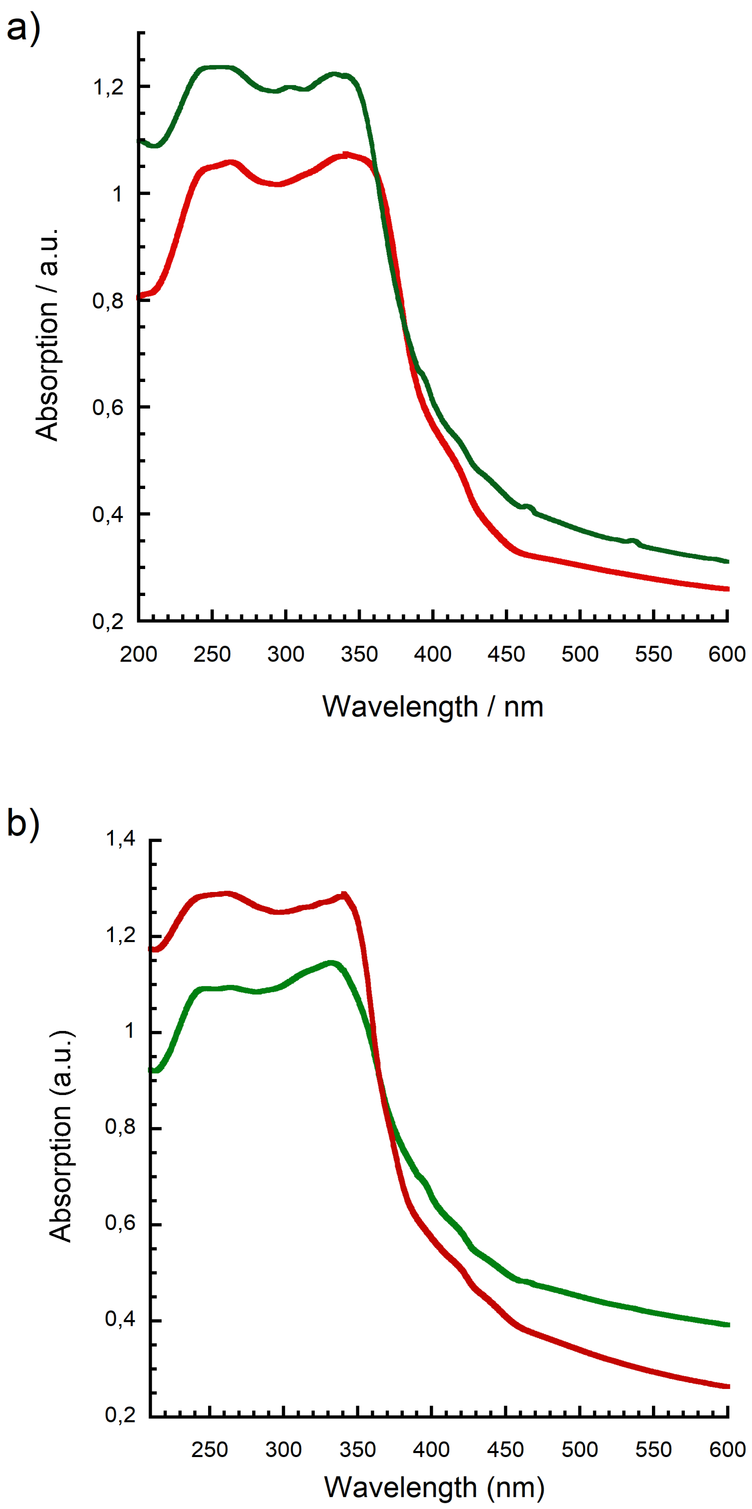

Figure S5. Electronic absorption spectra (green traces) of coordination polymers **1** (a) and **2** (b) compared to the spectra of their respective ligands $H_2\mathbf{L_1}$ and $H_2\mathbf{L_2}$ (red traces).

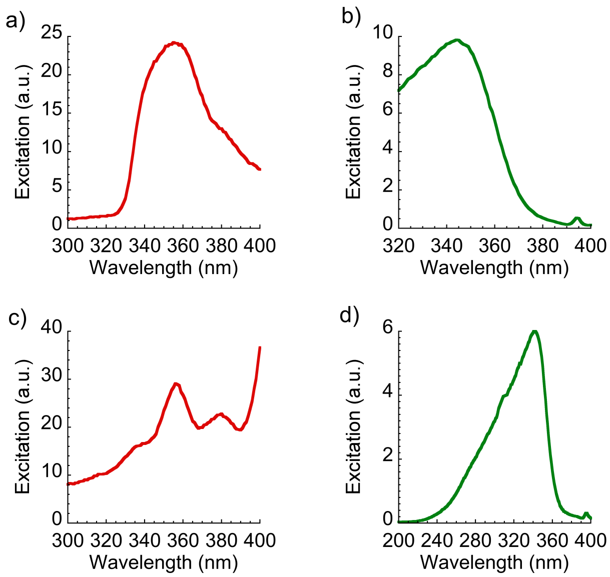

Figure S6. Excitation spectra of ligands $H_2\mathbf{L_1}$ (a, λ_{em} = 434 nm), $H_2\mathbf{L_2}$ (c, λ_{em} = 408 nm) and Eu^{3+} compounds **1** (b, λ_{em} = 614 nm) and **2** (d, λ_{em} = 614 nm).

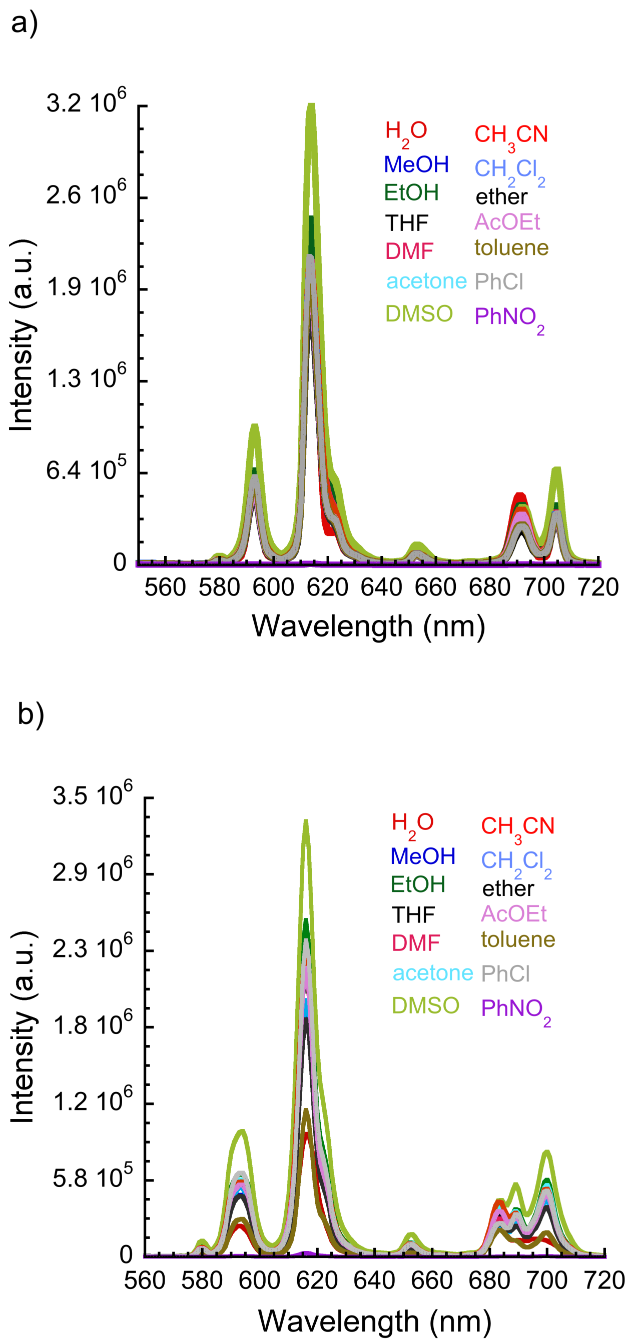

Figure S7. Emission spectra of **1** (a, λ_{exc} = 344 nm) and **2** (b, λ_{exc} = 340 nm) dispersed in different solvents.

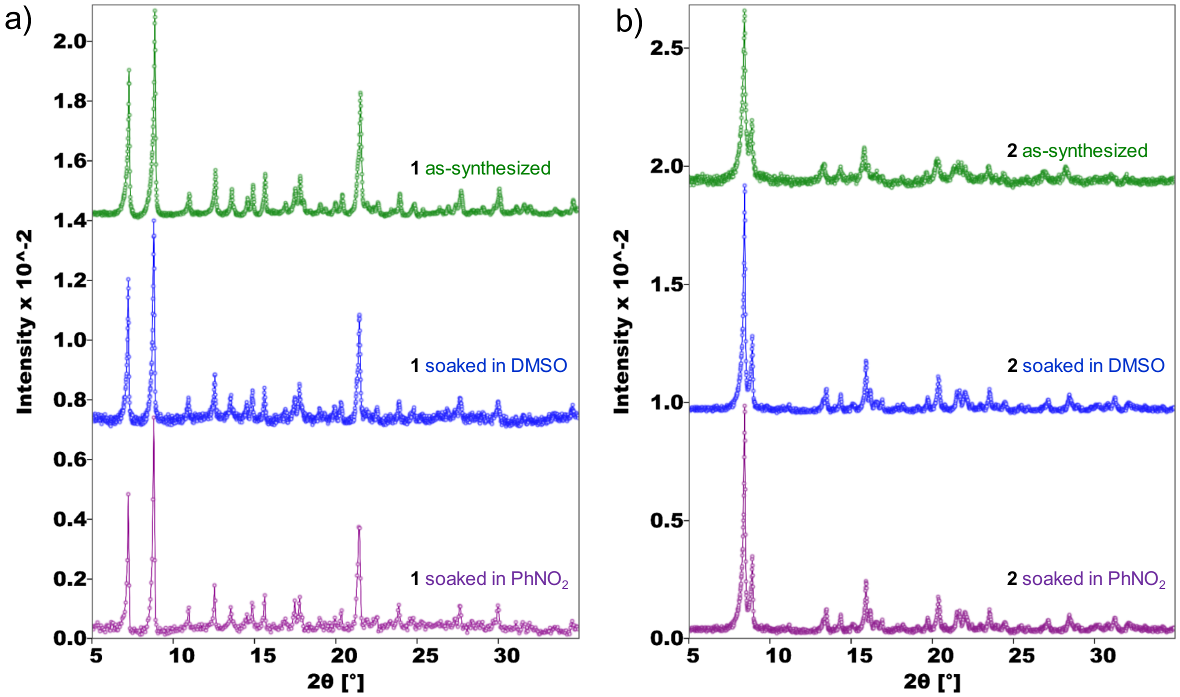

Figure S8. Powder X-ray diffractograms at room temperature of **1** (a) and **2** (b) as-synthesized and after soaking in DMSO and $PhNO_2$ (shown in green, blue and purple, respectively).

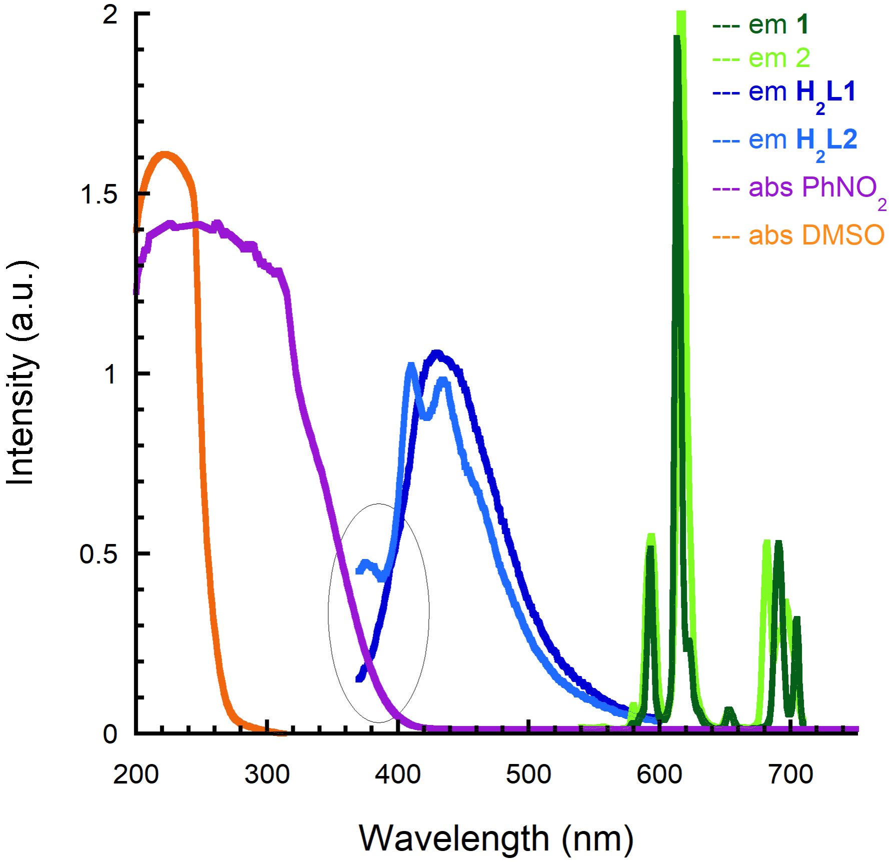

Figure S9. Comparison between the absorbance spectra of $PhNO_2$ and DMSO and the emission spectra of **1, 2**, $H_2\mathbf{L_1}$ and $H_2\mathbf{L_2}$.

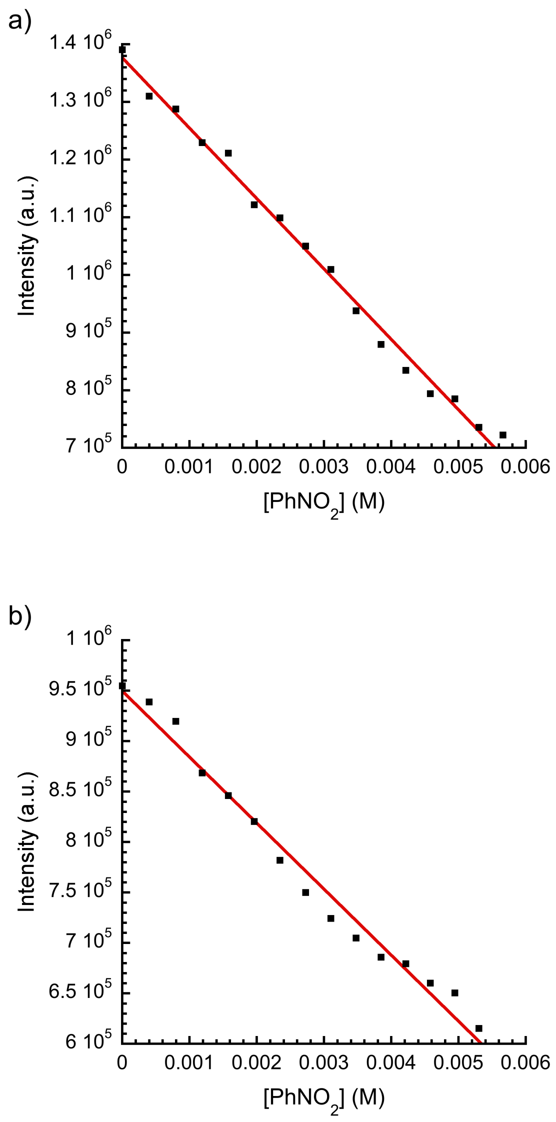

Figure S10. Variation of luminescence intensity of complexes **1** (a) and **2** (b) upon incremental addition of $PhNO_2$ at low concentrations. Best linear fits are shown in red.

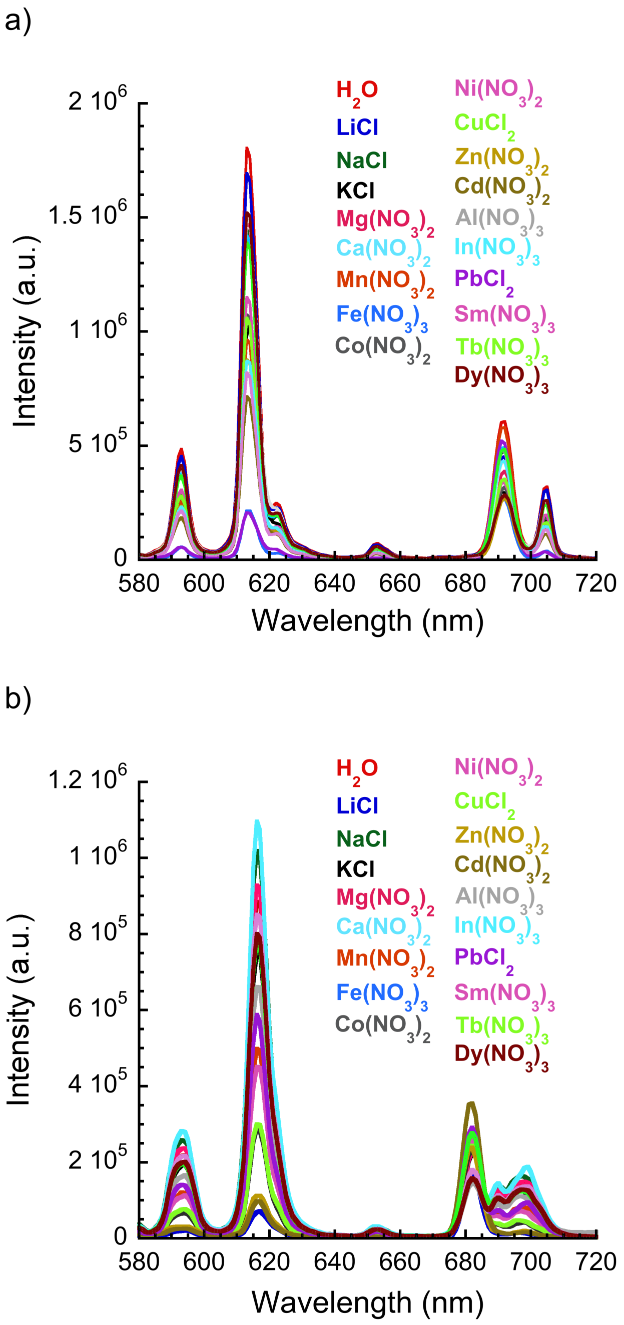

Figure S11. Emission spectra of **1** (a, λ_{exc} = 344 nm) and **2** (b, λ_{exc} = 340 nm) in 0.01 M aqueous solutions of different metal ions.

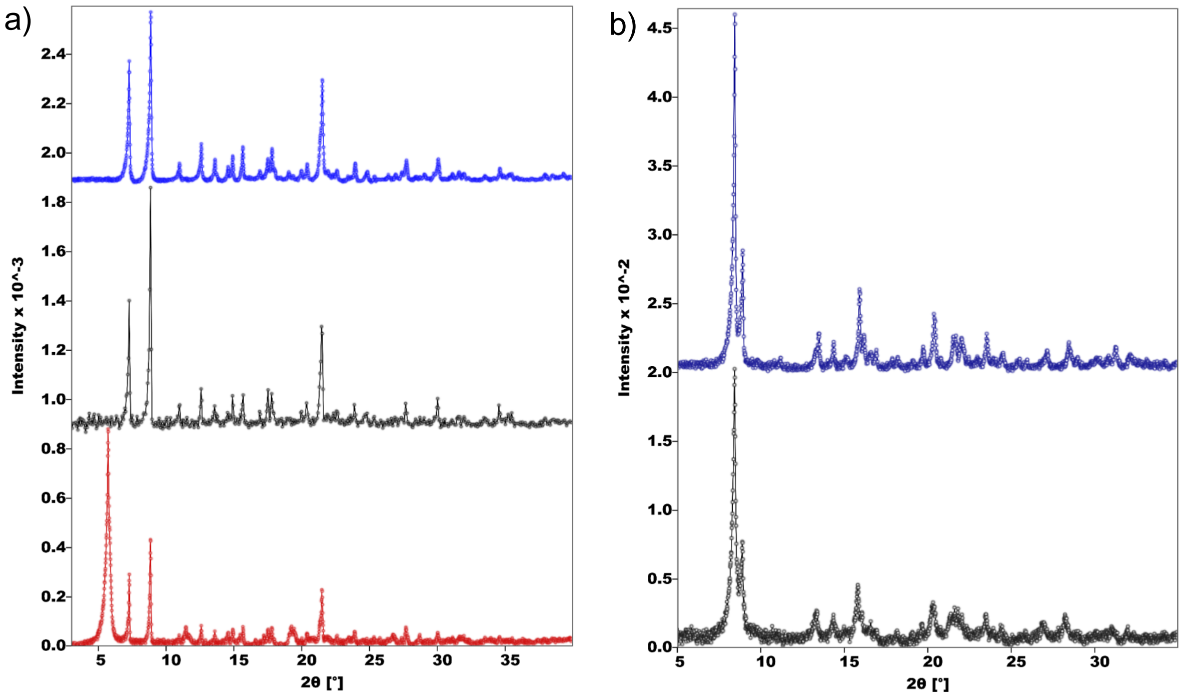

Figure S12. a) Powder X-ray diffractograms of **1** as-synthesised (blue) and after soaking in Fe^{3+} (black) and Pb^{2+} (red) aqueous solutions. b) Powder X-ray diffractograms of **2** as-synthesised (blue) and after soaking in a Fe^{3+} (black) aqueous solution.

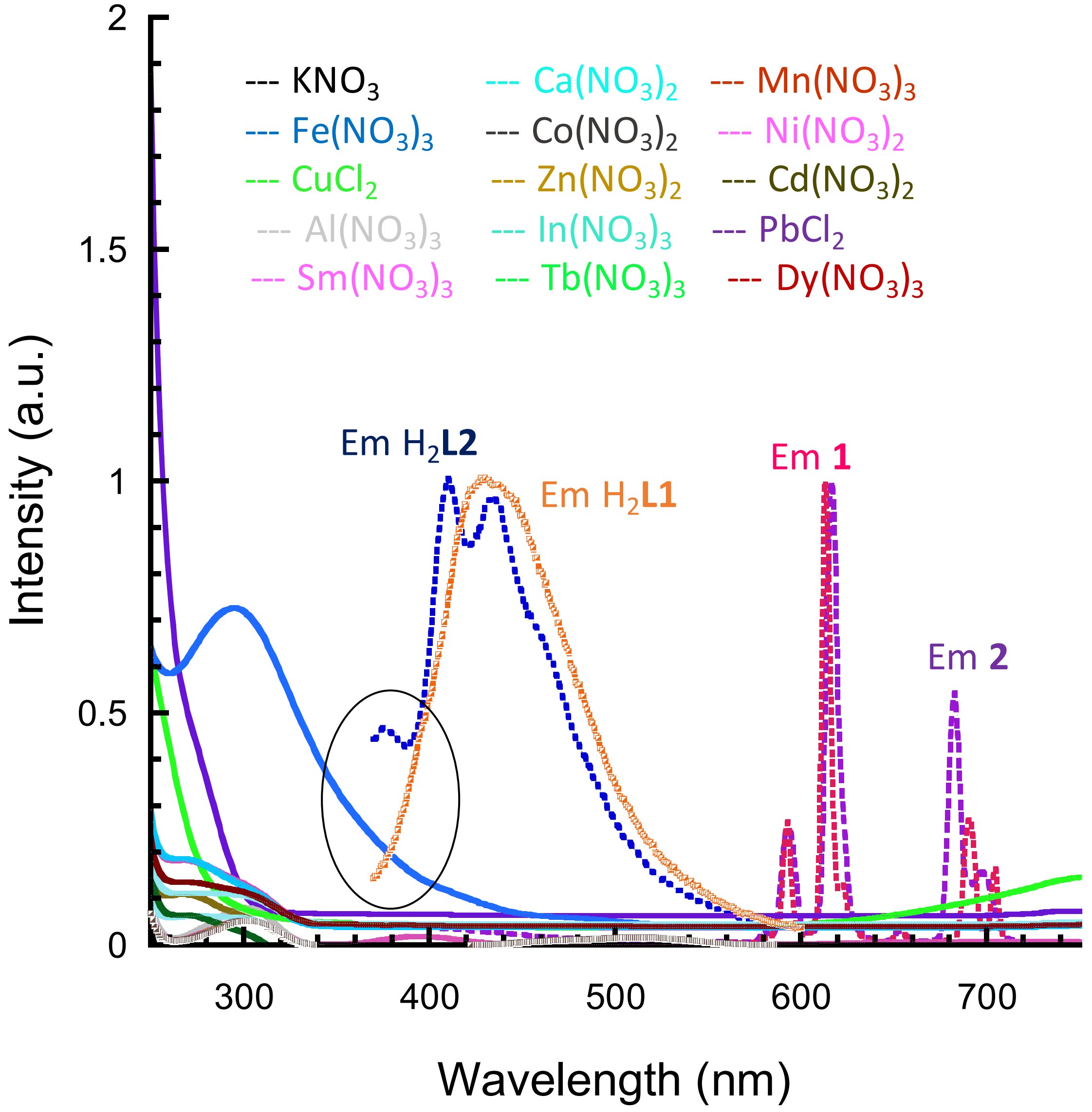

Figure S13. Electronic absorption spectra of 0.01 M aqueous solutions containing different metal ions for sensing experiments. Emission spectra of $H_2\mathbf{L_1}$, $H_2\mathbf{L_2}$, **1** and **2** are shown for comparison.

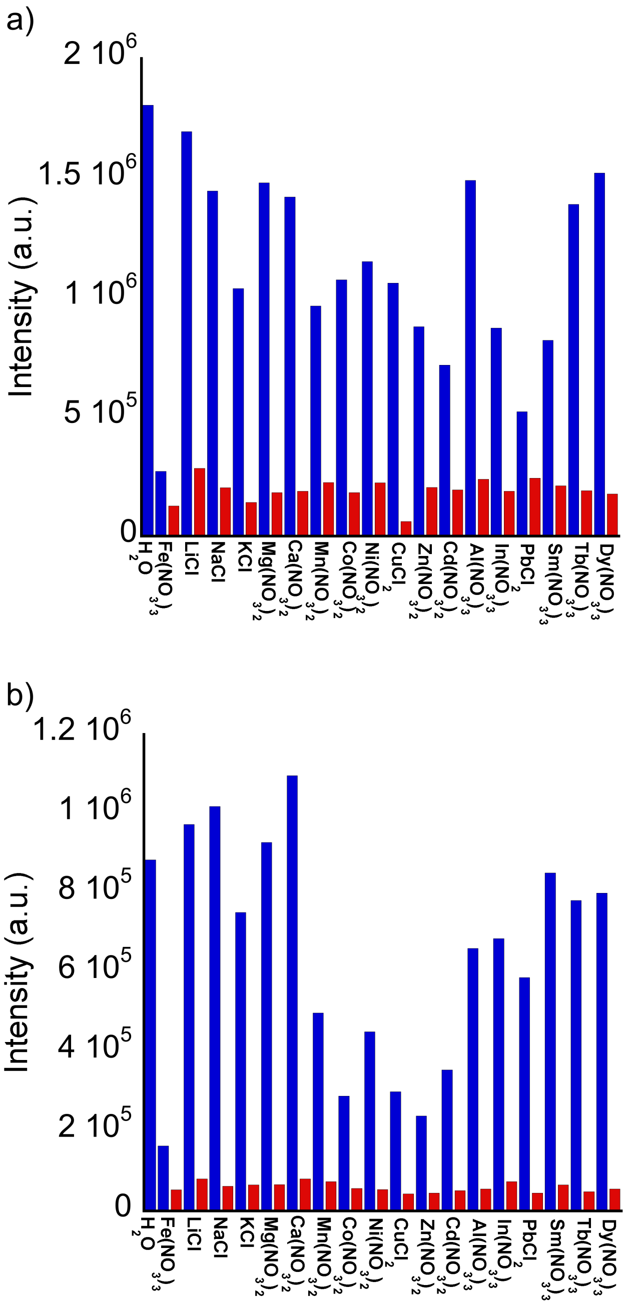

Figure S14. Comparison of the luminescence intensity (peak height at λ = 614 nm) of **1** (a) and **2** (b) dispersed in 0.01 M aqueous solutions of different metal ions (in blue) and dispersed in water with the addition of different ions (0.01 M) and Fe^{3+} (0.01 M) (in red).

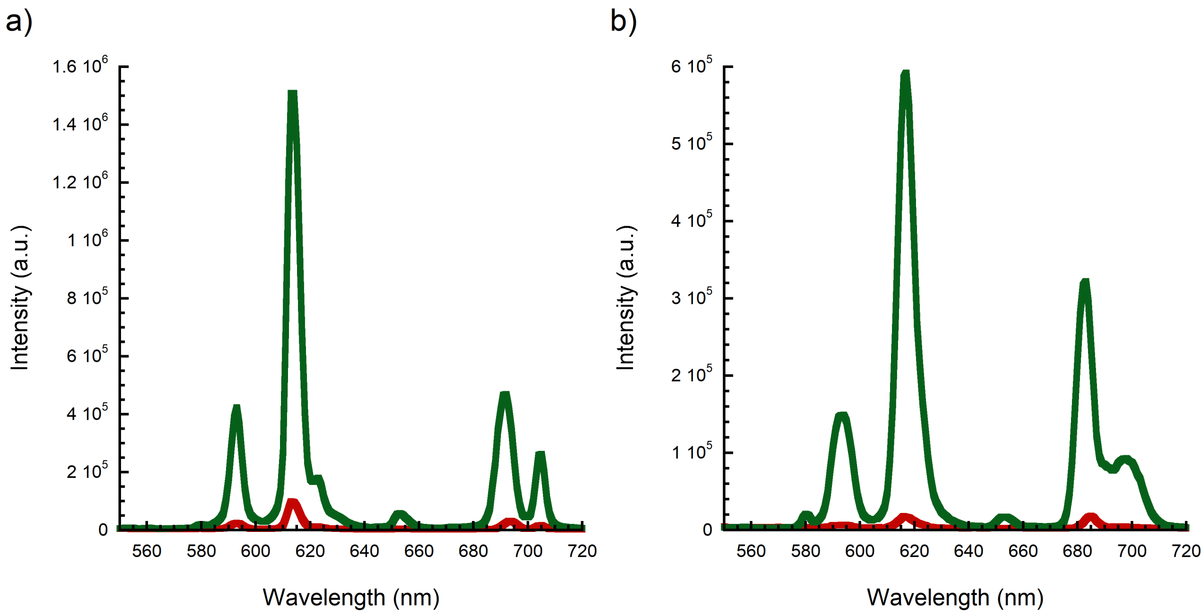

Figure S15. a) Emission spectra of suspensions of **1** prepared in 1 ml of water after addition of 600 μl of H_2O (green) and after addition of 598 μl of a 25 mM Fe^{3+} aqueous solution (red). b) Emission spectra of suspensions of **2** prepared in 1 ml of water after addition of 700 μl of H_2O (green) and after addition of 710 μl of a 50 mM Fe^{3+} aqueous solution (red).

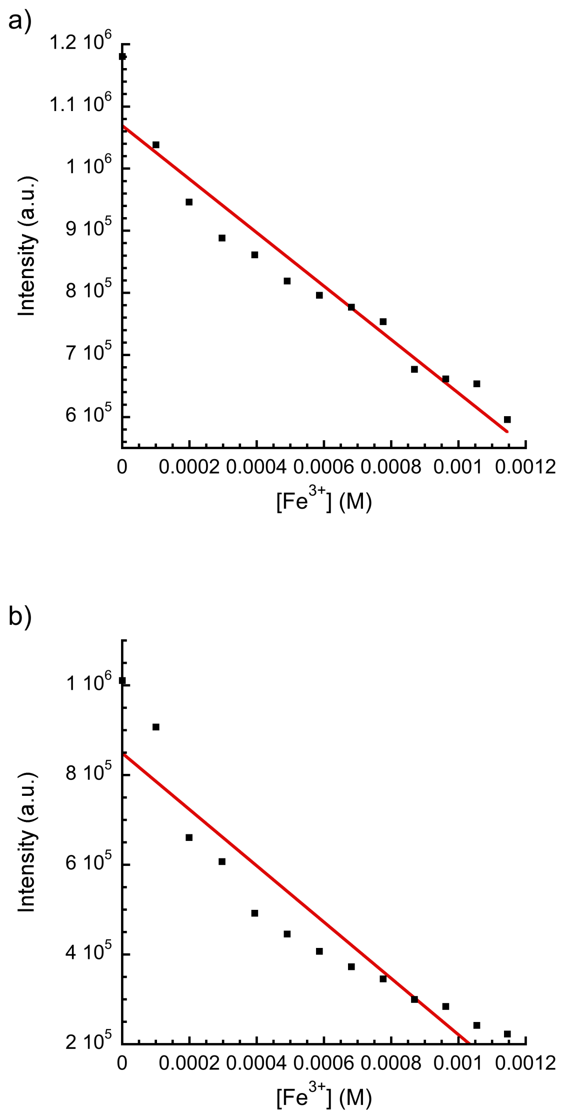

Figure S16. Variation of luminescence intensity of complexes **1** (a) and **2** (b) upon incremental addition of Fe^{3+} cations at low concentrations. Best linear fits are shown in red.

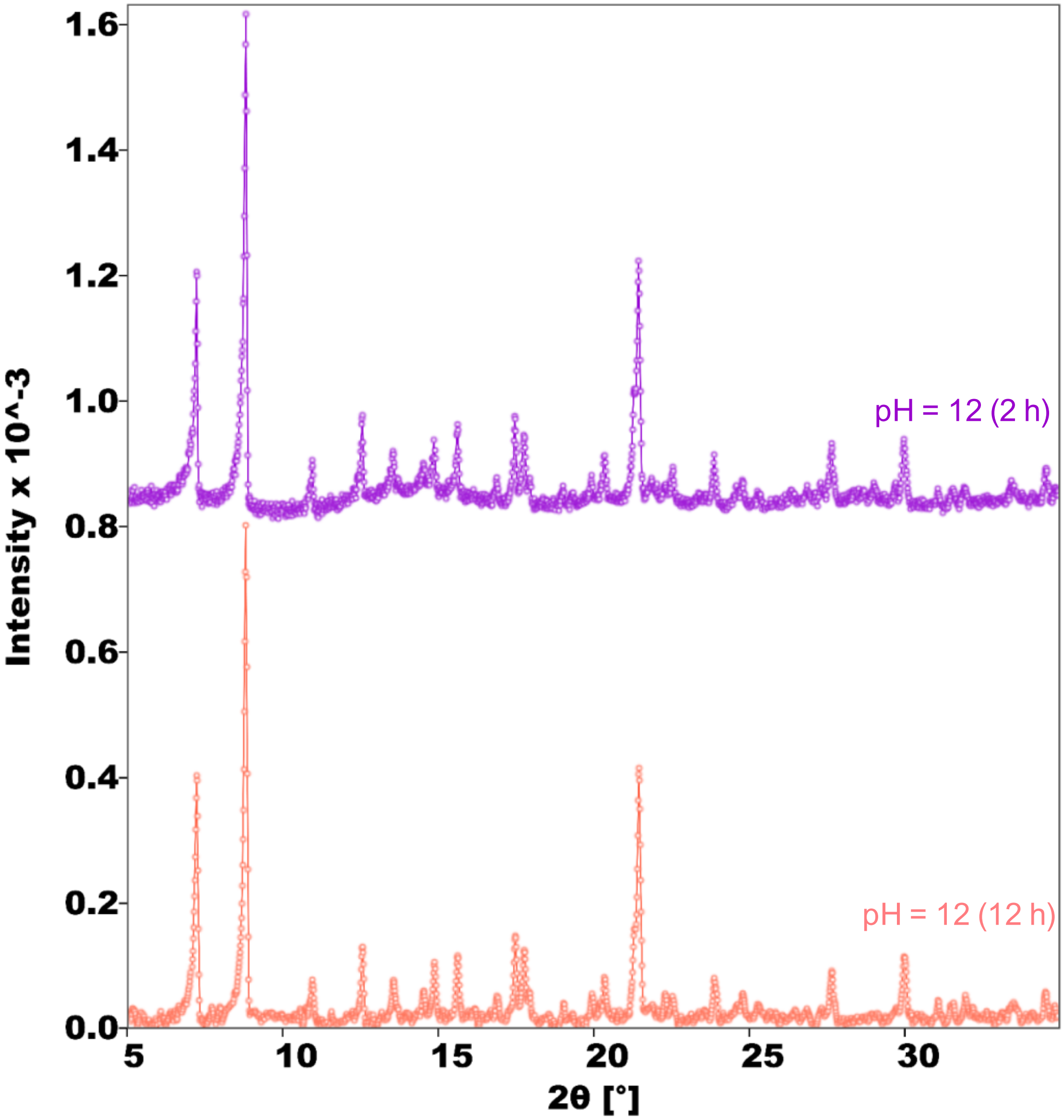

Figure S17. Powder X-ray diffractogram of **1** after soaking in an aqueous solution at pH = 12 for 2 h (purple) and 12 h (orange).

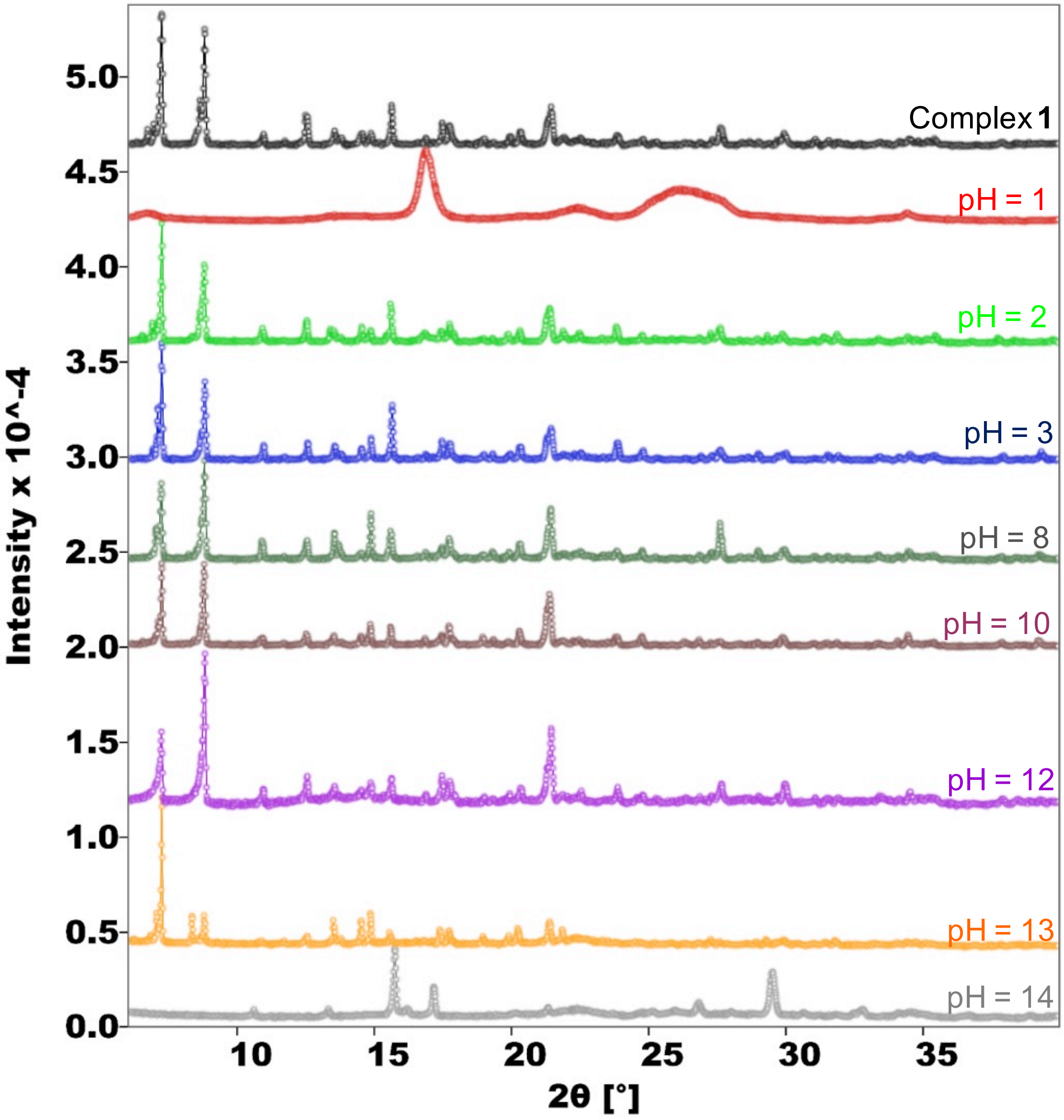

Figure S18. Powder X-ray diffractograms of **1** at different pH values.

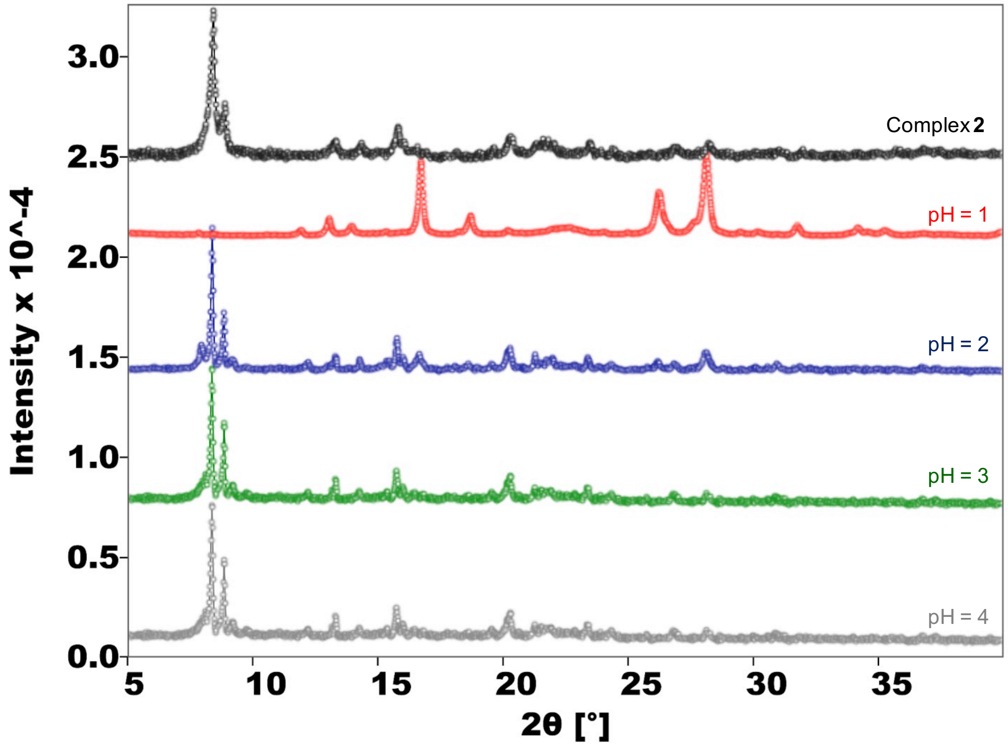

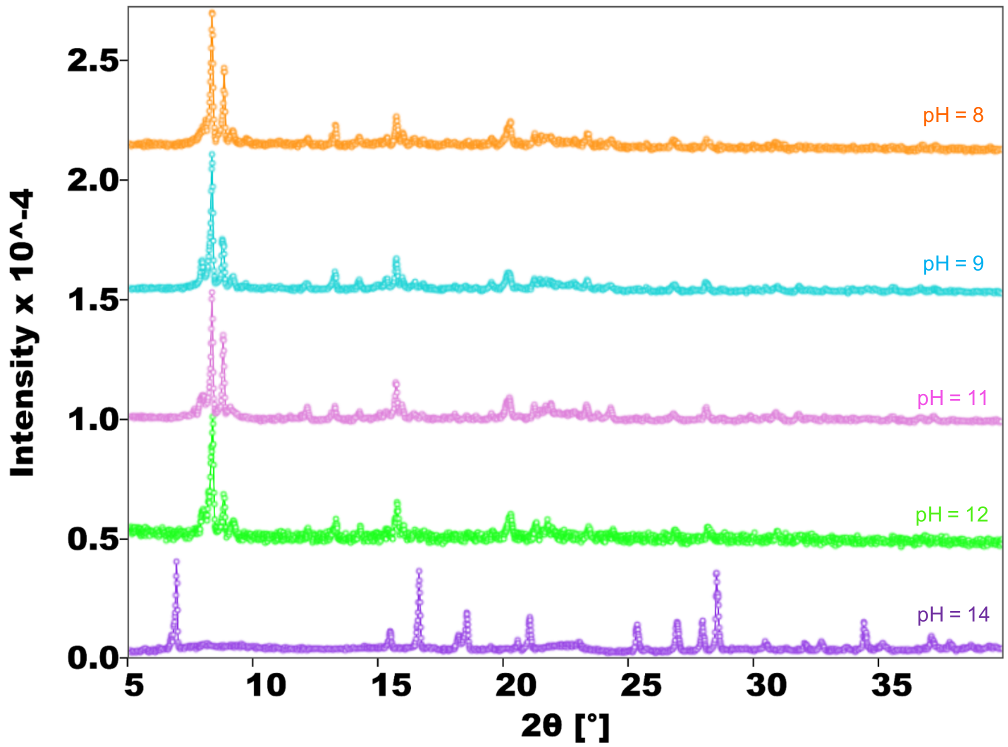

Figure S19. Powder X-ray diffractograms of **2** at different pH values.

Table S1. Stern-Volmer constants (K_{SV}) and limit of detection (LOD) of Fe^{3+} ions by luminescence quenching of europium coordination polymers in different solvents.

**Compound**	**Solvent**	**K_{SV} (M^{-1})**	**LOD (M)**
Eu^{3+}@MIL-124 [1]	H_2O	$3.87 \cdot 10^4$	$1 \cdot 10^{-2}$
$[Ln(Hpzbc)_2](NO_3)] \cdot H_2O$ [2]	EtOH		$1 \cdot 10^{-3}$
$[Eu(BTPCA)(H_2O)]$ [3]	DMF		$1 \cdot 10^{-5}$
$[Eu(H_2O)_2(BTMIPA)]$ [4]	DMF		$1 \cdot 10^{-5}$
$Eu(L)_3$ [5]	H_2O	$4.1 \cdot 10^3$	$1 \cdot 10^{-4}$
$[Eu_2(N\text{-}BDC)_3(DMF)_4]$ [6]	DMF	$2.88 \cdot 10^4$	
$[(CH_3)_2NH_2][Eu(C_{33}H_{24}O_{12})(H_2O)]$ [7]	H_2O	$3.874 \cdot 10^4$	$1 \cdot 10^{-2}$
$[Eu(HL')(H_2O)_2] \cdot 2H_2O$ [8]	H_2O		$1 \cdot 10^{-6}$
$[Eu(atpt)_{1.5}(phen)(H_2O)]$ [9]	EtOH	$7.60 \cdot 10^3$	$1 \cdot 10^{-3}$
$[(CH_3)_2NH_2][Eu(CPA)_2(H_2O)_2$ [10]	H_2O	$1.041 \cdot 10^4$	$1 \cdot 10^{-7}$
$[Eu(BPDA)_{1.5}] \cdot H_2O$ [11]	H_2O	$1.25 \cdot 10^4$	$1 \cdot 10^{-7}$
**1** (this work)	H_2O	$4.71 \cdot 10^2$	$5.82 \cdot 10^{-6}$
**2** (this work)	H_2O	$7.06 \cdot 10^2$	$3.16 \cdot 10^{-6}$

MIL-124: $Ga_2(OH)_4(C_9O_6H_4)$; H_2pzbc: 3-(1H-pyrazol-3-yl)benzoic acid; H_3BTPCA: 1,1,1-(benzene-1,3,5-triyl)tripiperidine-4-carboxylic acid; H_4BTMIPA: 5,5′-methylenebis(2,4,6-trimethylisophthalic acid); L: 4′-(4-carboxyphenyl)-2,2′:6′,2″-terpyridine; BDC: benzene-1,4-dicarboxylate; H_4L’: tetrakis[4-carboxyphenyl)-oxamethyl]methane acid; H_2atpt: 2-aminoterephthalic acid; phen: 1,10-phenanthroline; H_2CPA: 5-(4-carboxyphenyl)picolinic acid; BPDA: biphenyl-2,2’-dicarboxylic acid.

[1] Xu, X.-Y.; Yan, B. *ACS Appl. Mater. Interfaces* **2015**, *7*, 721. [2] Li, G.-P.; Liu, G.; Li, Y.-Z.; Hou, L.; Wang, Y.-Y.; Zhu, Z. *Inorg. Chem.* **2016**, *55*, 3952. [3] Tang, Q.; Liu, S.; Liu, Y.; Miao, J.; Li, S.; Zhang, L.; Shi, Z.; Zheng, Z. *Inorg. Chem.* **2013**, *52*, 2799 [4] Chen, Z.; Sun, Y.; Zhang, L.; Sun, D.; Liu, F.; Meng, Q.; Wang, R.; Sun, D. *Chem. Commun.* **2013**, *49*, 11557. [5] Zheng, M.; Tan, H.; Xie, Z.; Zhang, L.; Jing, X.; Sun, Z. *ACS Appl. Mater. Interfaces* **2013**, *5*, 1078. [6] Hao, J.-N.; Yan, B. *J. Mater. Chem. C* **2014**, *2*, 6758. [7] Dang, S.; Ma, E.; Sun, Z.-M.; Zhang, H. *J. Mater. Chem.* **2012**, *22*, 16920. [8] Liang, Y.-T.; Yang, G.-P.; Liu, B.; Yan, Y.-T.; Xi, Z.-P.; Wang, Y.-Y. *Dalton Trans.* **2015**, *44*, 13325. [9] Kang, Y.; Zheng, X.-J.; Jin, L.-P. *J Colloid Interf Sci.* **2016**, *471*, 1. [10] Wu, Y.-P.; Xu, G.-W.; Dong, W.-W.; Zhao, J.; Li, D.-S.; Zhang, J.; Bu, X. *Inorg. Chem.* **2017**, *56*, 1402. [11] Wang, J.; Wang, J.; Li, Y.; Jiang, M.; Zhang, L.; Wu, P. *New. J. Chem.* **2016**, *40*, 8600.

Table S2. Stern-Volmer constants (K_{SV}) and limit of detection (LOD) of nitrobenzene by luminescence quenching of coordination polymers in different solvents.

**Compound**	**Solvent**	**K_{SV} (M^{-1})**	**LOD (M)**
$[Eu(PBDC)_3(H_2O)_3]\cdot 2H_2O$[12]	H_2O	$3.00\cdot 10^3$	
$[Eu_3(PBDC)_4(H_2O)_4]\cdot 5H_2O$[12]	H_2O	$1.64\cdot 10^3$	
$[(CH_3)_2NH_2][Cd_2(L^{10})(DMA)]$[13]	DMA	$2.70\cdot 10^3$	$2.54\cdot 10^{-3}$
$[(N_3)Zn(L^{11})_2Zn]$[14]	DMF	$1.95\cdot 10^4$	$2.66\cdot 10^{-7}$
$[(SCN)Zn(L^{11})_2Zn]$[14]	DMF	$1.14\cdot 10^4$	$3.18\cdot 10^{-7}$
$[(SCN)Zn(L^{12})_2Zn]$[14]	DMF	$1.50\cdot 10^4$	$3.18\cdot 10^{-7}$
$[(N_3)Zn(L^{12})_2Zn]$[14]	DMF	$1.04\cdot 10^4$	$3.71\cdot 10^{-7}$
$[(N_3)Zn(L^{13})_2Zn]\cdot CH_2Cl_2$[14]	DMF	$0.94\cdot 10^4$	$3.56\cdot 10^{-7}$
$[Zn(H_2L^{14})(H_2O)]$ [15]	DMF	$3.26\cdot 10^3$	$7.2\cdot 10^{-6}$
$[Eu_2(pypymba)_6\cdot 4H_2O]\cdot 2H_2O$[16]	H_2O	$4.83\cdot 10^3$	$1\cdot 10^{-3}$
$[(CH_3)_2NH_2]_2[Eu_6(\mu^3\text{-}OH)_8(BPDC)_6(H_2O)_6]\cdot(solv)_x$[17]	H_2O	$5.42\cdot 10^3$	$4.06\cdot 10^{-6}$
**1** (this work)	H_2O	$1.50\cdot 10^2$	$2.05\cdot 10^{-5}$
**2** (this work)	H_2O	$1.60\cdot 10^2$	$3.03\cdot 10^{-5}$

H_2PBDC: 4'-(1H-pyrazol-3-yl)-[1,1'-biphenyl]-3,5-dicarboxylic acid; H_5L^{10}: 2,4-di(3′,5′-dicarboxylphenyl)benzoic acid]; H_2L^{11}: 2,20-[(1,3-propanediyl)bis(iminomethylene)]bis[6-ethoxyphenol]; H_2L^{12}: 2,20-[(2,2-dimethyl-1,3-propanediyl)bis(iminomethylene)]bis[6-methoxyphenol]; H_2L^{13}: 2,20-[(2,2-dimethyl-1,3-propanediyl)bis(iminomethylene)]bis[6-ethoxyphenol]; H_4L^{14}: 3-(3,5-dicarboxyphenyl)pyridine-2,6-dicarboxylic acid; DMA: N,N-dimethylacetamide; DMF: N,N-dimethylformamide; Hpypymba = 4-((3-(pyrazin-2-yl)-1H-pyrazol-1-yl)methyl)benzoic acid; H_2BPDC: biphenyl-4,4'-dicarboxylic acid.

[12] Li, H.; Han, Y.; Shao, Z.; Li, N.; Huang, C.; Hou, H. *Dalton Trans.* **2017**, *46*, 12201.

[13] Yan, Y.-T.; Liu, J.; Yang, G.-P.; Zhang, F.; Fan, Y.-K.; Zhang, W.-Y.; Wang, Y.-Y.; *CrystEngComm.* **2018**, *20*, 477.

[14] Karmakar, M.; Roy, S.; Chattopadhyay, S. *New. J. Chem.* **2019**, *43*, 10093.

[15] Zhao, Q.; Si, C.-D. *Cryst. Res. Technol.* **2019**, *54*, 1800155.

[16] Tang, Y.-Y.; Chen, S.; Wang, C.-J.; Zhu, Z.-X.; Liu, D.-N. *J. Coord. Chem.* **2018**, *71*, 3207.

[17] Gao, M.-L.; Cao, X.-M.; Zhang, Y.-Y.; Qi, M.-H.; Wang, S.-M.; Liu, L.; Han, Z.-B. *RSC Adv.* **2017**, *7*, 45029.

Table S3. Continuous shape measures (CShM) for **1** using SHAPE 2.1.

OP-8	Octagon (D_{8h})	20.295
HPY-8	Heptagonal pyramid (C_{7v})	17.911
HBPY-8	Hexagonal bipyramid (D_{6h})	21.291
CU-8	Cube (O_h)	16.960
**SAPR-8**	**Square antiprism (D_{4d})**	**9.458**
TDD-8	Triangular Dodecahedron (D_{2d})	10.575
JGBF-8	Johnson gyrobifastigium J26 (D_{2d})	22.600
JETBPY-8	Johnson elongated triangular bipyramid J14 (D_{3h})	24.654
JBTPR-8	Biaugmented trigonal prism J50 (C_{2v})	11.177
BTPR-8	Biaugmented trigonal prism (C_{2v})	11.166
JSD-8	Snub diphenoid J84 (D_{2d})	12.784
TT-8	Triakis tetrahedron (T_d)	17.043
ETBPY-8	Elongated trigonal bipyramid (D_{3h})	21.340

A CShM calculation of a molecular entity indicates its distance to an ideal shape, regardless of its size and orientation and is given by the following expression:

$$S(G) = min\frac{\sum_{k=1}^{N}|Q_k - P_k|}{\sum_{k=1}^{N}|Q_k - Q_0|} \cdot 100 \qquad \text{(S1)}$$

G is a specific and perfect symmetry group with coordinates P_k (k = 1, 2...N), whereas Q_k (k = 1, 2...N) indicates the coordinates of the N vertices of a given (experimental) structure. The distances between the vertices of the two objects are calculated until a set of P_k coordinates is found that minimises the distances (Q_0 is the coordinate vector of the centre of mass of the investigated structure and the denominator is a size normalisation factor). The lower the CShM parameter, the better the agreement between the investigated and reference coordination geometry. According to equation S1, CShM measures must lie within the range $100 \geq S \geq 0$. In compound **1**, a value of 9.458 still suggests a strong distortion from the ideal geometry. In fact, the skew angle (Φ), described as the offset between the two squares defined by the mean planes through the coordinated atoms, has a value of 37.7(4)°. This angle is quite different to that expected for an ideal D_{4d} symmetry (Φ = 45°).

Table S4. Continuous shape measures (CShM) for **2** using SHAPE 2.1.

EP-9	Enneagon (D_{9h})	29.326
OPY-9	Octogonal pyramid (C_{8v})	19.452
HBPY-9	Heptagonal bipyramid (D_{7h})	16.831
JTC-9	Johnson triangular cupola J3 (C_{3v})	14.994
JCCU-9	Capped cube J8 (C_{4v})	10.287
CCU-9	Spherical-relaxed capped cube (C_{4v})	10.776
JCSAPR-9	Capped square antiprism J10 (C_{4v})	4.614
CSAPR-9	Spherical capped square antiprism (C_{4v})	4.287
**JTCTPR-9**	**Tricapped trigonal prism J51 (D_{3h})**	**3.882**
TCTPR-9	Spherical tricapped trigonal prism (D_{3h})	4.037
JTDIC-9	Tridiminished icosahedron J63 (C_{3v})	14.392
HH-9	Hula-hoop (C_{2v})	10.860
MFF-9	Muffin (C_s)	4.345

Table S5. Values of quantum yield (PLQY) and lifetime (τ_0) for selected europium(III) complexes.

Compound	PLQY (%)	τ_0 (ms)
Eu-L1 [18]	32	1.25
[Eu(tta)$_4$(DEASPI)] [19]	28	
Eu-L2 [20]	12	1.65
Eu-L3 [20]	25	1.16
[Eu(dpq)(DMF)$_2$(NO$_3$)$_3$] [21]	32.5	0.47
[Eu(dppz)$_2$(NO$_3$)$_3$] [21]	38.5	0.49
Eu(PBI)$_3$·C$_2$H$_5$OH·H$_2$O [22]	26	0.25
Eu(PBI)$_3$·bpy [22]	68	0.98
Eu(PBI)$_3$·dmbpy [22]	72	1.18
Eu(PBI)$_3$·phen [22]	57	1.03
Eu(PBI)$_3$·bath [22]	71	1.01
Eu(MPPD)$_3$ [23]	8.2	0.12
Eu(MPPD)$_3$·bpy [23]	25.2	0.25
Eu(MPPD)$_3$·phen [23]	38.8	0.46
**1** (this work)	23	0.36
**2** (this work)	29	1.12

L1: 2,2'-(8-(3-N-maleimido)-1-carboxylatopropyl)-5,8,11-triaza-1,2,3(2,6)tripyridinacyclododecaphane-5,11-diyl)diacetate; tta: *trans*-4-[*p*-(N,N-diethylamino)styryl]-N-methylpyridinium tetrakis(2-thenoyltrifluoroacetonato); DEASPI: *trans*-4-[*p*-(N,Ndiethylamino)styryl]-N-methylpyridinium; L2: Pyridine-2,6-dicarboxylic acid; L3: Dipotassium salt of 4-(thien-2-yl)pyridine-2,6-dicarboxylic acid; dpq: dipyrido[3,2-d:2',3'-f]quinoxaline; dppz: dipyrido[3,2-a:2',3'-c]phenazine; dppz: dipyrido[3,2-a:2',3'-c]phenazine; HPBI: 3-phenyl-4-benzoyl-5-isoxazolone; bpy: 2,2'-bipyridine; dmbpy: 4,4'-dimethoxy-2,2'-bipyridine; phen: 1,10-phenanthroline; bath: 4,7-diphenyl-1,10-phenanthroline; MPPD: 1-(4-methoxyphenyl)-3-phenylpropane-1,3-dione.

[18] Faschinger, F.; Ertl, M.; Zimmermann, M.; Horner, A.; Himmelsbach, M.; Schöfberger, W.; Knör, G.; Gruber, H. J. ChemistryOpen. 2017, 6, 721-732.

[19] Shi, M.; Ding, C.; Dong, J.; Wang, H.; Tian, Y.; Hu, Z. Phys. Chem. Chem. Phys. 2009, 11, 5119-5123.

[20] Latva, M.; Takalo, H.; Mukkala, V.-M.; Matachescu, C.; Rodríguez-Ubis, J. C.; Kankare, J. J. Lumin. 1997, 75, 149-169.

[21] Dasari, S.; Patra, A. K. Dalton Trans. 2015, 44, 19844-19855.

[22] Biju, S.; Raj, D. B. A.; Reddy, M. L. P.; Kariuki, B. M. Inorg. Chem. 2006, 45, 10651-10660.

[23] Wang, D.; Zheng, C.; Fan, L.; Zheng, J.; Wei, X. Synthetic Met. 2012, 162, 2063- 2068.
